# Microbiota-Driven Immune Dysregulation Along the Gut–Lung–Vascular Axis in Asthma and Atherosclerosis

**DOI:** 10.3390/biomedicines14010073

**Published:** 2025-12-29

**Authors:** Elena-Larisa Zimbru, Răzvan-Ionuț Zimbru, Florina-Maria Bojin, Sorin Dan Chiriac, Laura Haidar, Minodora Andor, Gabriela Tănasie, Carmen Tatu, Marius Georgescu, Cristina Uța, Camelia-Felicia Bănărescu, Sabine Groza, Carmen Panaitescu

**Affiliations:** 1 Center of Immuno-Physiology and Biotechnologies, Department of Functional Sciences, “Victor Babes” University of Medicine and Pharmacy, 300041 Timisoara, Romania; elena.zimbru@umft.ro (E.-L.Z.); florinabojin@umft.ro (F.-M.B.);; 2 Research Center for Gene and Cellular Therapies in the Treatment of Cancer—OncoGen, Timis County Emergency Clinical Hospital “Pius Brinzeu”, No. 156 Liviu Rebreanu, 300723 Timisoara, Romania; 3 Timis County Emergency Clinical Hospital “Pius Brinzeu”, 156 Liviu Rebreanu Bd., 300723 Timisoara, Romania; 4 Discipline of Surgery III, Department X, Faculty of Medicine, “Victor Babes” University of Medicine and Pharmacy, 300041 Timisoara, Romania; 5 Multidisciplinary Heart Research Center, “Victor Babes” University of Medicine and Pharmacy, 300041 Timisoara, Romania; 6 Cardiology Clinic of the Timisoara Municipal Clinical Emergency Hospital, 12 Revolution of 1989 Bd., 300040 Timisoara, Romania

**Keywords:** microbiota, gut–lung–vascular axis, immune dysregulation, inflammation, allergic asthma, atherosclerosis, omics

## Abstract

**Background:** Asthma and atherosclerosis frequently coexist in clinical populations and share convergent immunometabolic pathways amplified by gut microbial dysbiosis. We propose the gut–lung–vascular axis as a unifying mechanistic framework connecting epithelial and endothelial inflammation providing a foundation for understanding shared inflammatory mechanisms beyond tissue-specific disease boundaries. **Methods:** A targeted narrative review systematically appraised clinical, experimental and multi-omics studies published over the last five years to delineate microbiota-driven pathways relevant to asthma and atherosclerosis. Particular emphasis was placed on specific microbial taxa, metabolite profiles and immunometabolic networks that connect gut dysbiosis with respiratory and cardiovascular dysfunction. **Results:** Across human and experimental cohorts, dysbiosis marked by depletion of short-chain fatty acids (SCFAs) producing taxa (*Faecalibacterium, Roseburia, Bacteroides*) and enrichment of pathobionts (Proteobacteria, *Haemophilus, Moraxella, Streptococcus*) promotes epithelial and endothelial barrier dysfunction, amplifying Th2/Th17-skewed inflammation and endothelial injury. Key metabolites, including SCFAs, trimethylamine N-oxide (TMAO), secondary bile acids (BA), indole/tryptophan derivatives and lipopolysaccharides (LPS), serve as molecular connectors linking gut, airway and vascular inflammation. Microbial signatures and metabolomic patterns hold emerging diagnostic and therapeutic potential, and several drug classes (e.g., statins, corticosteroids, proton-pump inhibitors (PPIs)) further modulate host–microbiota interactions. **Conclusions:** Shared microbial taxa and metabolite signatures in asthma and atherosclerosis support microbiota-mediated immune dysregulation along the gut–lung–vascular axis as a common pathogenic framework. Microbial and metabolite profiling may enable improved risk stratification and precise, microbiota-targeted therapies. Integrating microbiome-informed diagnostics and personalized interventions could help reduce systemic inflammation and the burden of these overlapping inflammatory diseases.

## 1. Introduction

Allergic asthma and atherosclerosis stand among the most prevalent chronic inflammatory diseases, each contributing significantly to global morbidity, mortality and economic burden [1,2]. Although asthma and atherosclerosis impact different organ systems, they share important immunological and pathological features, including persistent low-grade inflammation, activation of immune cells (mast cells, T cells), oxidative stress and endothelial dysfunction or epithelial barriers impairment [2,3]. Recent data, including research on microRNAs, suggest overlapping pathways that may mediate the link between respiratory and cardiovascular diseases, emphasizing the importance of targeted preventive measures and the development of personalized therapeutic strategies [4].

Beyond their individual impact, asthma and atherosclerotic cardiovascular disease (ASCVD) have been documented to co-occur within the same individuals. Clinical studies indicate that adults with persistent asthma exhibit higher rates of coronary heart disease, stroke and carotid atherosclerosis compared with non-asthmatic controls [2,3,5,6]. A recent systematic review and meta-analysis reported that individuals with asthma have a significantly elevated risk of composite cardiovascular disease and cardiovascular mortality, with summary relative risks around 1.3 for overall CVD and higher risks for specific outcomes such as angina pectoris, myocardial infarction and heart failure compared with non-asthmatic controls [7]. This augmented cardiovascular risk is particularly pronounced in adult-onset asthma and in women, supporting the existence of shared inflammatory and metabolic susceptibilities across these conditions [8].

Trillions of microorganisms in the gut perform fundamental metabolic and immunological functions, while dysbiosis has been associated not only with ASCVD but also with allergic asthma, suggesting that perturbations in gut microbial communities may drive systemic inflammation affecting both vascular and airway health [9]. We propose the concept of a gut–lung–vascular axis as a mechanistic framework linking asthma and atherosclerosis, suggesting that microbiome-driven immune dysregulation along this axis may contribute to the emergence of a novel, nontraditional cardiovascular risk factor, not yet integrated into current clinical paradigms. Therefore, the gut–X axis perturbations may constitute the initial modulation of pulmonary and vascular pathways, providing the systemic inflammatory background that drives the gut–lung–vascular axis [10]. The gut–X axis is at the core of bidirectional communication networks linking the gut microbiota with distant organs, such as the gut–lung, gut–brain and gut–vascular axes. These axes operate through coordinated immune, metabolic and neuroendocrine signaling pathways, and their disruption has been increasingly recognized as a contributor to the development and progression of chronic inflammatory and cardiometabolic diseases [11,12]. This conceptual framework emphasizes how microbiota-derived metabolites and immune mediators may act systemically to synchronize inflammatory responses across distant organs.

The human gut microbiota (GM) comprises approximately 3 × 10^13^ bacterial cells, predominantly belonging to taxa such as Bacteroidetes, Firmicutes, Actinobacteria, Proteobacteria, Fusobacteria, and Verrucomicrobia. These bacterial communities coexist and interact closely with other components of the gut ecosystem, including the mycobiota (fungae) and the viromes (mainly bacteriophages) [13]. Building upon this foundation, recent studies have introduced the concept of a “gut–vascular axis,” describing how gut microbial communities and their metabolites influence the development and progression of atherosclerosis [9,14,15]. Through intricate host–microbe interactions, the GM and its metabolic derivatives modulate vascular inflammation, endothelial integrity and function, as well as lipid metabolism, thereby preserving vascular health or facilitating atherosclerotic disease when disrupted [15,16].

Microbial communities extend beyond the well-known GM, colonizing other regions of the body, such as the oral cavity, skin, reproductive tract and respiratory tract, contributing to diverse functions in host regulation. These can also influence distal organs through systemic circulation, highlighting the interconnectedness of microbial ecosystems [17]. Despite differences in function, the gut and lungs share common developmental origins and mucosal architectures, with epithelial barriers and associated immune networks that maintain tissue homeostasis. Perturbations in the gut–lung axis can disrupt these networks, potentially exacerbating respiratory disease severity. In chronic respiratory conditions such as allergic asthma, the composition and function of the bacteriome, virome and mycobiome, along with their metabolites, are markedly altered compared to healthy individuals, although the precise mechanistic pathways remain incompletely defined [18]. Emerging evidence indicates that gut microbial dysbiosis may enhance susceptibility to allergic airway diseases via the gut–lung axis. Characterizing these host–microbiota interactions offers opportunities for the identification of disease biomarkers and the development of microbiome-targeted therapies for allergic asthma [19,20].

Disturbances in microbial composition and function, collectively referred to as dysbiosis, have been implicated in a wide spectrum of chronic inflammatory and metabolic disorders [15,19,21,22,23]. Emerging evidence emphasizes the role of microbiota-derived metabolites, such as short-chain fatty acids (SCFAs) and trimethylamine N-oxide (TMAO), in modulating systemic inflammation, vascular integrity and immune regulation [24,25,26]. The concept of the gut–lung and gut–vascular axis has become increasingly widespread, suggesting that dysbiosis in one compartment can exert distant effects on other organ systems [10,15,24,27,28].

A comprehensive literature analysis was performed using PubMed. To ensure methodological rigor and reflect current conceptual frameworks, the search focused on studies published within the preceding five years. Eligible sources included clinical, experimental and omics studies addressing host–microbiota interactions and immune signaling networks in asthma and atherosclerosis. Particular emphasis was placed on dysbiosis-related inflammation, alterations in microbial metabolites such as SCFAs and TMAO, and their subsequent effects across the gut–lung–vascular crosstalk.

This review synthesizes recent evidence revealing the immunometabolic interplay between allergic asthma and atherosclerosis through microbiota-driven mechanisms. By integrating insights from metagenomic, metabolomic and immunologic studies, we propose the gut–lung–vascular axis as a novel conceptual framework explaining how dysbiosis contributes to systemic inflammation and multi-organ pathology. Therapeutic strategies aimed at modulating the intestinal microbiome, through diet and lifestyle changes, targeted microbial supplementation, reducing environmental stressors, may be promising approaches for improving systemic inflammation and enhance clinical outcomes in both cardiovascular and respiratory diseases. Understanding this interconnected axis may guide precision strategies for microbiome-based prevention and therapy.

## 2. Microbiota: An Overview

**Figure 1:**
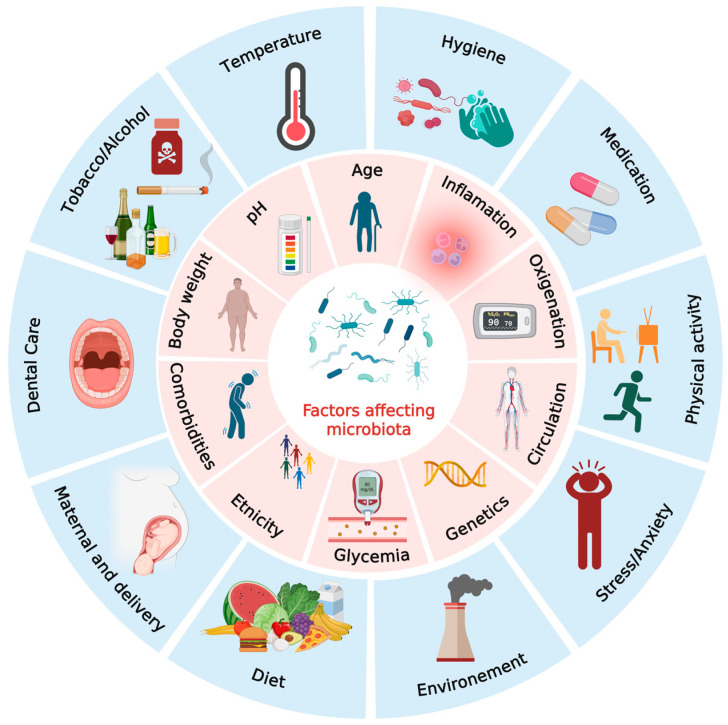
Major factors influencing gut microbiota composition and function. This figure illustrates the multifactorial determinants shaping the gut microbiota throughout life. These interconnected factors determine the composition, metabolic output and resilience of the gut microbiota, ultimately impacting host immunity, metabolism and disease susceptibility. Created in BioRender. Zimbru, E. (2025) https://BioRender.com/27osdfr.

Interactions between the host and its microbiota are essential for maintaining physiological balance [29]. Also, microbial metabolites, particularly SCFAs, support regulatory T-cell differentiation and anti-inflammatory pathways, while commensal species reinforce epithelial and endothelial barrier function [30,31]. When this equilibrium is disrupted, a state of dysbiosis arises, fostering systemic inflammation that can drive the development of diseases such as allergic asthma and atherosclerosis [24,32,33]. In asthma, microbial imbalance contributes to airway hyperresponsiveness and enhanced immune activation, whereas in atherosclerosis, it promotes endothelial dysfunction, immune cell recruitment and increased production of pro-inflammatory cytokines [28,34,35]. The gut microbiota, representing about 70% of the human microbial population, shows marked variation along the digestive tract due to chemical and immunological gradients [17,36,37]. Colonization begins at birth, shaped by delivery mode, breastfeeding and early-life exposures and shifts from facultative to strict anaerobes as the host matures [38,39]. The human microbiota is primarily composed of five dominant bacterial phyla: Firmicutes, Bacteroidetes, Proteobacteria, Actinobacteria and Verrucomicrobia [29,40,41,42,43]. Within these groups, Firmicutes (approximately 60–80%) include the classes Clostridia, Bacilli and Negativicutes, while Bacteroidetes (roughly 20–40%) encompass Bacteroidia, Flavobacteria, Sphingobacteria and Cytophagia, consisting exclusively of Gram-negative taxa. Generally, strict anaerobes such as *Bacteroides*, *Clostridioides*, *Eubacterium*, *Ruminococcus*, *Peptococcus*, *Fusobacterium* and *Bifidobacterium* dominate over facultative anaerobes including *Lactobacillus*, *Escherichia*, *Enterobacter*, *Enterococcus*, *Proteus* and *Klebsiella* [44,45]. Other phyla, such as Cyanobacteria, Fusobacteria and Spirochaetaceae, are present at lower abundance within the human microbial ecosystem [45,46]. Gut microbiota composition is influenced by host genetics and physiology, environment, diet, chronic disorders, medication use, physical activity, stress, circadian rhythm, habits (alcohol or tobacco use), maternal microbiota, early-life microbial encounters, hygiene, urban versus rural living and exposure to animals, with diet exerting the strongest impact through its effects on microbial diversity and metabolite production (Figure 1) [44,47,48,49,50,51,52,53,54].

### 2.1. Diet

Dietary composition represents one of the principal determinants of gut microbiota architecture and metabolic function, acting as a major factor governing host–microbiome homeostasis. Sustained dietary patterns modulate microbial diversity, metabolic function and inflammatory balance, thereby exerting profound and long-term effects on host physiology and susceptibility to chronic conditions [47,54].

The Mediterranean diet, characterized by high consumption of fiber-rich plant foods, legumes, polyphenol-containing fruits, whole grains, fish and extra-virgin olive oil, consistently promotes microbial diversity and metabolic resilience. Individuals following a Mediterranean diet typically exhibit increased abundances of *Bacteroides*, *Prevotella*, *Lactobacillus*, *Faecalibacterium*, *Clostridioides* and *Oscillospira*. This microbial pattern is associated with a higher Bacteroidetes-to-Firmicutes ratio and greater alpha-diversity, reflecting a more balanced and resilient gut ecosystem. The beneficial effects are largely attributed to the diet’s high fiber and unsaturated fatty acid content, which favor saccharolytic and anti-inflammatory bacterial communities [55,56,57].

In contrast, Western-style diets, typically high in saturated fats, refined carbohydrates, animal protein and food additives, are associated with reduced microbial diversity, increased Firmicutes and a decline in *Bacteroides* and expansion of pathobionts such as Enterobacteriaceae, *Bilophila wadsworthia* and *Alistipes* spp. Such dysbiotic configurations favor the production of pro-inflammatory metabolites (e.g., TMAO, lipopolysaccharides (LPS)) that impair gut barrier function and activate toll-like receptor-mediated inflammatory signaling, promoting metabolic endotoxemia and insulin resistance [58,59]. Similarly, high-fat/high-fructose diets have been shown in animal and human studies to disrupt intestinal tight-junction integrity, suppress SCFA synthesis and elevate oxidative stress, collectively contributing to obesity-associated inflammation and vascular dysfunction [60,61].

Other dietary patterns also elicit distinct microbial responses. Plant-based diets, encompassing vegetarian and vegan regimens, increase the abundance of fiber-degrading and saccharolytic taxa such as *Prevotella* and *Ruminococcus*, thereby enhancing SCFA output and reducing intestinal pH. The resulting metabolic environment supports colonocyte health and anti-inflammatory immune regulation [62]. In contrast, ketogenic diets, while clinically beneficial for metabolic disorders, are linked to decreased microbial diversity and reduced levels of *Bifidobacterium* and *Eubacterium rectale*, accompanied by elevated bile-acid metabolism and inflammatory gene expression [63,64].

Low-fiber diets negatively affect gut microbial composition by depleting fermentable substrates for beneficial taxa such as *Faecalibacterium prausnitzii* and *Roseburia*. Sustained fiber deficiency fosters dysbiosis, increasing susceptibility to metabolic and inflammatory disorders, including obesity, diabetes and atherosclerosis [65,66,67].

Diets high in fermented foods enhance gut microbial diversity and promote beneficial taxa such as *Lactobacillus* and *Bifidobacterium*. Their bioactive metabolites support epithelial integrity, increase SCFA production and reduce systemic inflammation. Regular consumption of fermented foods like yogurt, kefir, sauerkraut, pickles, sourdough bread or fermented beverages has been linked to improved immune regulation and metabolic health [68,69].

### 2.2. Medication Use

Pharmaceutical agents profoundly influence gut microbial composition and function, often producing long-lasting effects on host metabolism and immunity [70].

Recent evidence indicates that pharmacological treatments, including corticosteroids and antibiotics (used in asthma exacerbation treatment), can notably alter GM composition and metabolic activity. Statins, such as atorvastatin and rosuvastatin, have been shown to reshape gut microbial profiles by increasing beneficial taxa such as *Bifidobacterium longum*, *Akkermansia muciniphila* and *Ruminococcus obeum*, while reducing potentially pathogenic species like *Parabacteroides merdae* [71,72]. These changes may enhance SCFA production and bile acid metabolism, contributing to the anti-inflammatory and lipid-lowering effects of statins, while interindividual differences in microbiota composition may explain variable therapeutic responses [72,73]. Such disruptions in microbial diversity and metabolite production (e.g., SCFAs, secondary bile acids, TMAO, LPS) have been linked to impaired gut-barrier integrity, immune dysregulation and altered drug metabolism, ultimately influencing both disease progression and therapeutic efficacy [25,31,74].

Many routinely prescribed medications induce distinct shifts in gut microbial ecology, with some taxa, such as *Streptococcus salivarius*, increasing across multiple drug classes, while others displaying drug-specific signatures, including *Bifidobacterium dentium* with PPIs, *Clostridium leptum* with tricyclic antidepressants, *Eubacterium ramulus* with selective serotonin reuptake inhibitors (SSRIs) and a decrease in *Bacteroides fragilis* with metformin [75]. Other agents, such as steroid inhalers, statins, methotrexate, laxatives and L-thyroxine, also remodel microbial composition and functional pathways in predictable patterns [75].

Antibiotics remain the most potent disruptors, reducing microbial diversity, depleting beneficial taxa such as *Bifidobacterium* and *Faecalibacterium prausnitzii* and promoting the overgrowth of opportunistic pathogens and antibiotic-resistant strains. Broad-spectrum antibiotics have a very potent iatrogenic disruptor effect of the gut ecosystem, producing acute and occasionally long-lasting reductions in microbial diversity, loss of key commensals and changes in microbiome functional capacity. By eliminating susceptible taxa, they create microbial micro-environments that favor the overgrowth of opportunistic pathogens such as *Clostridioides difficile*, with varied clinical consequences that may result in life-threatening colitis [76,77]. Moreover, even short antibiotic courses can lead to persistent alterations in microbial metabolic capacity and resilience [78,79].

More broadly, metagenomic studies demonstrate that almost one-quarter of non-antibiotic medications exert antibiotic-like effects, suppressing gut bacterial strains and reshaping microbial composition [70].

Corticosteroids also modify the gut ecosystem by suppressing immune-regulated microbial niches and favoring the proliferation of pathobionts, contributing to increased intestinal permeability and systemic inflammation [80]. Oral glucocorticoid therapy markedly induces alterations in the gut microbiome, characterized by increased richness but reduced overall diversity. It leads to a higher Firmicutes/Bacteroidetes ratio, a signature frequently associated with metabolic dysregulation and induced consistent shifts at the genus level, including reductions in beneficial taxa such as *Bifidobacterium*, *Faecalibacterium*, *Akkermansia* and *Prevotella*, alongside a proliferation of *Streptococcus*, *Collinsella* and *Parabacteroides* [81]. Functionally, oral glucocorticoids impaired considerably the gut’s capacity to generate short-chain fatty acids, as confirmed by a reduced expression of key SCFA-producing enzymes and increased gut serotonin levels, reflecting broad metabolic rewiring [81]. It remains uncertain to what extent the inhaled corticosteroids reshape the diversity of specific bacterial taxa in the airways or in the gut, in the latter likely via the swallowed drug fraction and its systemic effects [73]. In severe asthma, high-dose inhaled corticosteroids modulate the pathological immune activation and airway inflammation. Their pharmacologic effects are associated with an enrichment of Proteobacteria and a reduced abundance of Bacteroidetes, *Prevotella* and Fusobacteria in the gut [82]. In parallel, a balanced intestinal microbiota has a reciprocal influence upon the immune cell activation, as outer membrane proteins from gut microbiota promote the formation and functionality of Tregs while inhibiting excessive Th2 and Th17 cell activation [83,84]. Consequently, the gut microbiota is essential for preserving immune homeostasis primarily by counterbalancing the detrimental impact of Th2/Th17 hyperactivation and beneficial effects of Tregs responses [83].

Proton-pump inhibitors (PPIs) are recognized as major and usually under-appreciated promoters of gut dysbiosis. Their use is associated with reduced bacterial abundance and changes involving up to 20% of detectable taxa, resulted from altering gastric pH and facilitating the survival and translocation of oral bacteria to the gut and increasing the abundance of *Enterococcus*, *Streptococcus* and *Escherichia coli*, which have been linked to dysbiosis-related disorders [85,86,87]. Sometimes, the microbiome impact of PPIs could exceed that of antibiotics or other common drugs [85].

Additionally, cardiovascular drugs such as statins, beta-blockers, ACE inhibitors and platelet aggregation inhibitors can modulate gut microbial metabolism, influencing bile acid transformation, SCFA production, TMAO and microbial diversity [73]. Altered gut microbiota and their associated metabolites are closely connected to the pathogenesis of atherosclerosis, dyslipidaemia, hypertension and heart failure, indicating that gut bacteria could biotransform cardiovascular drugs, consequently modifying their pharmacodynamics, pharmacokinetics and toxicity. For statins, pretreatment levels of microbiota-derived bile acids reliably predict the extent of LDL-cholesterol lowering, reflecting a bidirectional relationship in which microbial communities both influence and are modulated by lipid-lowering therapy [73,88]. In atherosclerosis, gut dysbiosis is marked by depletion of SCFA-producing *Faecalibacterium* and *Roseburia* and increase in *Streptococcus*, *Escherichia* and other opportunistic pathogens, enhancing TMAO production, NLRP3–IL-1 activation and endothelial dysfunction [40]. Statins can remodel gut microbial communities, typically shifting towards more SCFA-producing and BA-transforming taxa, while also lowering pro-atherogenic TMAO generating bacteria and inflammatory markers. Conversely, baseline dysbiosis characterized by reduction of SCFA-producing genera and a proliferation of pathobionts has been linked to attenuated LDL-cholesterol reduction and sustained vascular inflammation under statin treatment. Further supporting this, higher levels of SCFA-producing gut microbiota were reported during effective statin therapy [40,72,89]. Therefore, lipid control alone might not be a fully effective preventive strategy in some cases of atherosclerosis [40]. Furthermore, statin treatment in mice led to increased blood glucose levels and body weight, accompanied by specific alterations in the metabolic profile of the gut microbiota [72,75,90]. This pattern would indicate that lipid-lowering medications are partly microbiota-dependent and that the gut microbiota is both a target and a determinant of statin efficacy.

### 2.3. Age

Across the human lifespan, the gut microbiota experiences continuous and finely regulated transitions that mirror developmental, metabolic and immunological changes. The gut microbiome undergoes predictable transitions, from rapid colonization and ecological instability in infancy, through relative equilibrium in adulthood and finally to compositional decline in elderly [38,91,92,93]. Advancing age, is associated with a progressive decline in community diversity, reduced abundance of short-chain fatty acid-producing taxa and an increase in pro-inflammatory bacteria, fostering low-grade systemic inflammation and metabolic dysfunction, characteristic of “inflammaging” [94,95]. Large-scale cohort analyses have further linked these compositional shifts with frailty, immune dysregulation and cardiometabolic disease [96]. Evidence from studies of healthy aging populations indicates that gut microbiome decline is not inevitable. A large Chinese cohort found that healthy older adults, even beyond 94 years, shared microbiome profiles similar to young adults [97]. Centenarians often display higher microbial diversity, enriched in beneficial taxa such as *Akkermansia*, Christensenellaceae, Lachnospiraceae and Ruminococcaceae, suggesting links between microbial richness, resilience and longevity [98,99]. In contrast, frailty and cognitive decline are associated with reduced diversity, loss of *Faecalibacterium prausnitzii* and increased abundance of proinflammatory taxa including *Eggerthella* and *Ruminococcus gnavus* [100,101]. Despite these trends, inconsistencies across studies emphasize the observational nature of most microbiome-aging research and the need for mechanistic insight into how specific microbial shifts influence health outcomes in older adults [94].

### 2.4. Host Genetics and Physiology

Host genetic variation influences gut microbiota via bile acid metabolism. A murine study identified the *Slc10a2* transporter as a key link between bile acid regulation and microbial composition [102]. Gut microbes transform primary bile acids into diverse metabolites that influence host signaling and microbial ecology [103]. The gut microbiome is partly regulated by host genetics, with heritability estimates ranging from 5–45%, though more recent studies suggest that only 3–13% of microbial taxa are heritable, indicating that genetics accounts for only a small fraction of inter-individual microbiome variation [104,105]. Twin and genome-wide studies have identified numerous host loci and at least 30 genes have been experimentally validated to influence microbial composition. Genetic variants can affect disease either directly, by altering host physiology, or indirectly, through microbiome-mediated mechanisms, emphasizing the need for integrative research linking host genetics, microbiota and disease phenotypes [104]. Although genetic effects are modest compared to environmental influences, they define a physiological framework within which diet, immunity and microbial ecology interact to maintain host-microbe homeostasis [106]. Probiotics can regulate miRNA expression, influencing immune balance and inflammation. The proposed microbiota–miRNA–lung axis suggests that gut microbes may epigenetically affect lung health. Combining probiotics with miRNA-modulating therapies (agomiRs and antagomiRs) could enhance anti-inflammatory effects, but more studies are needed to confirm these mechanisms and therapeutic benefits [4,107].

### 2.5. Maternal Microbiota and Early-Life Microbial Exposures

The maternal microbiota profoundly modulates neonatal immune ontogeny, with microbial transfer during gestation contributing to the programming of immune tolerance and predisposing or protecting against allergic asthma in later life [39]. Early-life microbial exposures, including delivery mode, breastfeeding and antibiotic use, further modulate immune tolerance, with dysbiosis in infancy predisposing individuals to chronic inflammation and heightened cardiovascular risk [38,91]. During vaginal birth, infants acquire microbes primarily from the mother’s vaginal and intestinal flora, whereas those delivered by C-section are mainly colonized by skin-associated bacteria [108]. Moreover, C-section and subsequent colonization with *Clostridioides difficile* have been linked to increased asthma risk, whilst vaginally born children were only pre-disposed if a parent suffered from an atopic disease. Probiotic supplementation has shown potential as an adjunctive approach for asthma prevention and symptom control [109,110]. Although defining a universal core respiratory microbiota remains challenging due to substantial individual variability, several genera, including *Staphylococcus*, *Streptococcus*, *Haemophilus*, *Moraxella* and *Veillonella*, are consistently detected within the respiratory tract [111].

Early-life gut and airway microbiota shape immune development and influence susceptibility to allergic asthma or ACVDs later in life [38,40,112]. Infants with reduced *Bifidobacterium*, *Akkermansia*, *Faecalibacterium* or fungal genera such as *Candida* and *Rhodotorula* are at higher risk of developing allergic diseases, whereas the presence of *Faecalibacterium*, *Lachnospira*, *Veillonella* or *Rothia* supports immune tolerance and modulates asthma risk [34,112,113]. These findings emphasize the importance of early microbial exposures and suggest that targeted interventions, dietary, probiotic and/or prebiotic, beneficially reshape the GM, enhance SCFAs synthesis and ameliorate systemic inflammation, providing potential benefits for both asthma management and vascular health.

### 2.6. Environmental Exposures

Environmental exposures such as hygiene practices, urban versus rural living and interactions with animals strongly shape early microbial colonization. For example, infants raised in rural households or on farms show greater colonization by beneficial bacteria like *Bifidobacterium*, *Lactobacillus* and *Bacteroides*, as well as higher diversity of anaerobic commensals, which is associated with lower allergy risk [114]. In contrast, children growing up in urban environments exhibit less microbial richness, altered gut and airway microbiota and increased susceptibility to asthma and sensitization [115]. Moreover, household exposures to animals and animal fecal contamination further modify the gut microbiome and resistome in early childhood, especially in less-sanitized environments [116]. A prospective analysis of over 200,000 participants from the UK Biobank found that urban environment features (air pollution, complex street networks) were associated with greater ASCVD risk, partly mediated by adverse cardiometabolic status and mental health pathways [117]. Additionally, recent studies indicate that higher levels of ambient particulate matter (PM_2.5_), nitrogen dioxide (NO_2_) and road traffic noise are linked to increased incident CVD risk, emphasizing these as key environmental risk factors [118,119].

### 2.7. Chronic Conditions

Recent evidence highlights the microbiota contribution to the pathogenesis of numerous chronic diseases, including autoimmune disorders, inflammatory diseases, metabolic syndromes and cancer, through its intricate crosstalk with the host immune system [120]. Dysbiosis has been associated with increased inflammatory responses and impaired metabolic regulation, as observed in some diseases such as type 2 diabetes and obesity [121]. Moreover, microbiota composition can influence tumor initiation, progression and therapeutic outcomes [122]. Modulation of gut microbial communities, through probiotics, prebiotics or bioactive compounds like phenolics, represents an emerging therapeutic strategy aimed at restoring eubiosis and improving clinical outcomes in complex chronic diseases [123,124].

### 2.8. Lifestyle Factors

Lifestyle factors significantly influence the gut microbiota: for example, higher levels of physical activity have been associated consistently with increased microbial diversity and favorable changes in microbial composition in numerous human cohorts [125,126]. Disruption of circadian rhythms and sleep–wake cycles alters the diurnal dynamics of microbial communities and has been shown to impair gut barrier function and microbial-derived metabolite production [127]. Habitual behaviors such as excessive alcohol consumption and smoking are linked to decreased microbial diversity, increased conditionally pathogenic abundance and heightened intestinal permeability, thus promoting dysbiosis and inflammation [128,129]. Physiological stress exerts pronounced effects on the gut microbiota through activation of the hypothalamic–pituitary–adrenal (HPA) axis, sympathetic nervous system and associated hormonal mediators, which in turn alter gut motility, secretions and barrier integrity [130]. In humans, higher levels of psychological stress are associated with reductions in microbial abundance and diversity, characterized by a reduction in beneficial genera such as *Lachnospira*, *Sutterella*, Lachnospiraceae, *Phascolarctobacterium* and *Veillonella*, alongside an increased prevalence of taxa including *Methanobrevibacter*, *Rhodococcus* and *Roseburia* [130,131]. Animal models further reveal that stress-induced increases in glucocorticoids and catecholamines promote shifts toward potentially pathogenic taxa and increased intestinal permeability [132,133,134]. Emerging evidence shows that microbiota changes can influence stress responses by altering HPA axis activity, creating a bidirectional gut–brain feedback loop under prolonged stress conditions [135].

## 3. Microbiota in Allergic Asthma

### 3.1. Evidence for the Gut–Lung Axis in Asthma

In recent years, the hypothesis of the existence of a "gut–lung axis" has become increasingly relevant in explaining the pathogenic mechanisms of asthma, pointing to the complex and bidirectional interaction between intestinal and pulmonary immunity [19,35,42,83]. Recent data suggest that alterations in the composition of the intestinal microbiota can influence inflammatory processes in the airways through microbial metabolites, bacterial components (such as endotoxins) and systemic immune regulation [136,137]. Prospective studies in infants have shown that delayed or abnormal development of the intestinal microbiota in the first years of life correlates with an increased likelihood of childhood asthma [112,138].

Studies have shown that fermentation of dietary fiber by Lactobacillaceae and Bifidobacteriaceae increases SCFAs, which suppress Th2-type inflammation partly via Treg induction [139,140]. *Lactobacillus* and *Bifidobacterium* enhance IL-10 and inhibit IgE-dependent responses, while probiotics also modulate Th17 pathways. DCs conditioned with *Lactobacillus reuteri* or *Lactobacillus casei* promote Th1 polarization and Treg expansion [141,142]. Probiotic use has likewise been associated with increased IFN-γ [143].

At the same time, analysis of the respiratory microbiome has shown notable differences between asthmatic patients and healthy individuals, indicating that dysbiosis in the respiratory tract may contribute to the perpetuation of chronic lung inflammation [43,144]. Taken together, this evidence supports the existence of a functional connection in the gut–lung axis, in which microbial communities at these levels interact and influence each other in the development of allergic respiratory diseases.

### 3.2. Microbial Taxa Associated with Allergic Asthma

- Airway Microbiome

Changes in the composition and diversity of the airway microbiota are now widely recognized as important features of allergic asthma, reflecting both disease-related dysbiosis and immune–microbial interactions. The phylum Proteobacteria, with prominent genera such as *Haemophilus* and *Moraxella*, is often overrepresented in the respiratory tract of individuals with asthma. These taxa have been linked to epithelial barrier disruption and reduced responsiveness to corticosteroid therapy [144]. Certain members of the Firmicutes phylum, including *Streptococcus* species, may influence airway immune tone and inflammatory signaling, though their impact appears to vary depending on strain-specific characteristics and local microenvironmental conditions. In contrast, commensal Actinobacteria, notably *Corynebacterium* and *Dolosigranulum*, are associated with a stable, health-related airway microbiome. Their early colonization in infancy has been correlated with a lower risk of respiratory infections and allergic airway disease later in life, suggesting a protective role in maintaining epithelial and immune homeostasis [43].

- Gut Microbiome

The gut microbiota exerts a profound influence on immune homeostasis and allergic sensitization. Reduced abundance of Bacteroidetes in infancy has been consistently linked to an increased risk of asthma development [31]. In contrast, Clostridia (class Firmicutes), including genera such as *Faecalibacterium* and *Roseburia*, are known producers of SCFAs that enhance regulatory T-cell function and promote immune tolerance [138]. These compositional patterns suggest that both local (airway) and distal (gut) microbial shifts are intertwined in shaping asthma susceptibility and phenotype expression.

Co-sensitization to ragweed pollen and house dust mites induces a more severe allergic asthma phenotype, especially in animals receiving a high-fructose diet, which exacerbates systemic inflammation, obesity, dyslipidemia and airway hyperresponsiveness. These changes are commonly associated with diet-induced dysbiosis and metabolic imbalance [145].

### 3.3. Mechanisms: Immune Modulation and Metabolite Signaling

Dysbiosis may disrupt immune homeostasis by promoting Th2 polarization (via increased IL-4, IL-5, IL-13 production) and diminishing regulatory responses. Conversely, specific commensals or their metabolites enhance Treg differentiation and suppress effector T-cell activation [146]. Dendritic cells and innate lymphoid cells (ILC2s) are also modulated by microbial signals, influencing antigen presentation, cytokine release and epithelial crosstalk [71].

Microbial metabolites are key mediators of the gut–lung axis.

- SCFAs such as acetate, propionate and butyrate are fermentation products of dietary fibers by Clostridia species. SCFAs enhance Treg expansion, suppress airway eosinophilia and modulate gene expression through histone deacetylase (HDAC) inhibition and G-protein-coupled receptor (GPR41, GPR43, GPR109A) activation [146]. Early-life exposure to propionate, including through breast milk, has been associated with reduced airway inflammation, while short-chain fatty acids modulate immune responses through HDAC and activation of G-protein-coupled receptors [147,148,149]. Recent studies also show that butyrate can selectively inhibit Tfh13 cells involved in allergic sensitization [136,148,149].
- Tryptophan metabolites, particularly indole derivatives, act as ligands for the aryl hydrocarbon receptor (AhR), promoting epithelial integrity and limiting allergic inflammation [31]. Recent studies in asthma show that type-2 inflammation perturbs tryptophan metabolism and that exogenous indoles restore microbial diversity, reduce OVA-IgE/cytokines and ameliorate airway disease in vivo through AhR-dependent signaling [150].
- Secondary bile acids generated by gut microbial metabolism may also exert systemic immunoregulatory effects, though their contribution to asthma pathophysiology remains less well characterized [138]. Secondary BA supplementation has been shown to reduce allergen-driven airway inflammation, but evidence in human asthma remains limited and inconsistent [28].

### 3.4. Microbiota-Targeted Interventions: Probiotics, Prebiotics and Dietary Modulation

Emerging therapeutic strategies aim to restore microbial balance and enhance host-microbe interactions.

- Probiotics: Supplementation with *Lactobacillus*, *Bifidobacterium*, *Lachnospira* or *Akkermansia* strains has shown potential to reduce airway inflammation and improve asthma control, though clinical results remain heterogeneous and strain-specific [151]. The PRObiotics in Pediatric Asthma Management (PROPAM) study evaluated the efficacy of a probiotic formulations containing *Ligilactobacillus salivarius* LS01 + *Bifidobacterium breve* B632. A reduction in both the incidence and severity of asthma exacerbations was observed among the children receiving probiotic supplementation [152]. Multistrain formulations containing *Lactobacillus* and *Bifidobacterium* species have been shown to enhance asthma symptom control and modulate inflammatory biomarkers, although their impact on lung function parameters remains variable across studies [151]. Recent review on gut–lung axis modulation identifies *Lachnospira* as a short-chain fatty acid-producing genus associated with protective, anti-inflammatory effects in asthma, though direct clinical evidence from supplementation trials remains limited [19].
- Prebiotics and dietary modulation: High-fiber diets enhance SCFA production, improve mucosal immune tolerance and reduce airway hyperresponsiveness in animal models [19]. In murine allergic asthma models, a high-fiber diet (or cellulose-enriched diet) attenuated airway inflammation and symptoms, altered gut microbial composition toward beneficial taxa and in some cases increased SCFAs [153]. Human observational data associate higher fiber intake with reduced biomarkers of airway inflammation and better asthma control, though prospective evidence is limited [154]. Increasing the intake of fermentable foods are being explored as adjuncts in asthma therapy with the aim of modulating the gut–lung axis via microbiota and metabolite pathways [19,155]. Higher fiber intake during mid-childhood appears to be associated with a lower risk of allergen sensitization later in life, although part of this association may reflect reduced exposure to dietary allergens due to food avoidance in sensitized individuals [154]. Dietary interventions targeting microbiota composition are being actively explored as adjunctive strategies for asthma management.
- Advanced approaches: Innovative microbiome-based approaches, including fecal microbiota transplantation (FMT) and helminth-derived immunomodulators, have recently attracted interest as potential adjunctive strategies for restoring immune balance in allergic asthma. Experimental studies indicate that transferring gut microbial communities from healthy donors can ameliorate airway hyperresponsiveness, reduce eosinophilic infiltration and normalize intestinal microbial diversity in murine asthma models, largely through the enhancement of SCFAs synthesis and expansion of regulatory T-cell populations [156]. Clinical evidence remains preliminary, with only limited human data and no proven efficacy in asthma management. Research on helminth-derived molecules shows immunoregulatory activity through suppression of Th2/Th17 inflammation, induction of IL-10-producing Tregs and modulation of TLR-mediated signaling [157,158].

Microbiota-based strategies hold promise for complementing standard asthma therapies by re-establishing immune balance along the gut–lung axis. However, further randomized controlled trials are needed to determine optimal microbial strains, dosage, patient phenotype, regiotype or endotype and treatment duration for clinical benefit.

## 4. Microbiota in Atherosclerosis

### 4.1. Role of the Gut Microbiota in Cardiovascular Disease

In recent years, mounting evidence has implicated the intestinal microbiome as a significant modulator of CVD, including the development and progression of atherosclerosis. The “gut–heart axis” concept emphasizes that alterations in gut microbial ecology may influence vascular health through metabolic, inflammatory and immune-mediated pathways [159].

Several human cohort studies and animal investigations demonstrate that dysbiosis, for example shifts in the relative abundance of major phyla such as Firmicutes versus Bacteroidetes, or expansion of potentially pathogenic taxa is associated with increased markers of subclinical atherosclerosis and coronary artery disease [160,161,162].

The gut microbiota may not only modulate classical risk factors (hypertension, dyslipidemia, diabetes mellitus and/or obesity) but may act directly on the vascular wall and atherogenic process [163].

### 4.2. Microbial Metabolites Implicated in Atherosclerosis

One of the central mechanistic links between the gut microbiome and atherosclerosis is the generation of biologically active microbial metabolites that enter the systemic circulation and act on vascular or immune targets.

- SCFAs (acetate, propionate and butyrate) are major fermentation products of dietary fiber in the gut [164]. SCFAs exert pleiotropic cardiovascular effects, improving endothelial function and blood pressure control while attenuating systemic inflammation [164,165]. Recent studies indicate that increasing SCFAs levels attenuate risk factors for atherosclerosis by promoting eubiosis and reinforcing intestinal barrier integrity [164,165,166]. Dysregulation of acetyl-CoA metabolism, essential for SCFA synthesis, has been reported in hypertensive individuals, whereas *Bacteroides acidifaciens*-derived acetate and propionate have shown cardioprotective effects [167,168]. Likewise, butyrate-producing bacteria such as *Roseburia intestinalis* reduce vascular inflammation and ACVD by preserving mucosal integrity and limiting systemic endotoxin translocation [169,170]. SCFAs act primarily via G-protein-coupled receptors (OLFR78, GPR41 and GPR43) that regulate vascular tone and immune balance [148,171]. Propionate signaling through GPR41/OLFR78 elicits antihypertensive effects, whereas butyrate and acetate enhance nitric oxide bioavailability, supporting endothelial homeostasis [171,172,173]. High dietary fiber intake correlates with lower blood pressure and improved vascular outcomes in humans, in part due to the anti-inflammatory activity of SCFAs [174]. Butyrate maintains epithelial barrier integrity and inhibits histone deacetylases, leading to epigenetic suppression of pro-inflammatory mediators, thereby reducing cytokines such as IL-1β and TNF-α in experimental models [175,176,177]. However, excessive circulating SCFA levels, especially under high-protein/high-fiber diets, have been associated with unfavorable lipid profiles, indicating that SCFA effects depend on dietary composition and microbial context [178].
- TMAO represents one of the most extensively characterized gut–vascular co-metabolites [179]. Formed from choline, L-carnitine and phosphatidylcholine via microbial trimethylamine (TMA) synthesis and hepatic oxidation, elevated TMAO levels consistently correlate with higher atherosclerotic burden and cardiovascular risk [163,179]. Meta-analyses of large cohorts (>26,000 participants) reveal a dose-dependent association between TMAO and adverse cardiovascular outcomes [179,180]. Experimental evidence demonstrates that transplanting TMAO-producing microbiota into ApoE−/− mice accelerates plaque development, while TMAO suppression reverses these effects [181,182]. TMAO formation is diet-dependent, with omnivorous diets producing more TMAO than vegetarian or vegan diets [182]. Mechanistically, TMAO promotes atherosclerosis through foam cell formation, platelet hyperreactivity and increased inflammatory signaling [164,177,179]. Elevated TMAO levels associate with upregulated C-reactive protein, IL-1β and vascular nuclear factor kappa B (NF-κB) activation, contributing to plaque instability [177,179,183]. Microbial enzyme TMA lyase inhibition has shown promise in reducing TMAO-driven vascular injury [184,185].
- Bile acids (BAs) are another class of microbiota-influenced metabolites. They are saturated or hydroxylated steroids that aid absorption of dietary fats, lipophilic vitamins and metabolic regulation of lipids, glucose and systemic metabolic signaling [163,164,186]. Gut microbiota convert hepatic primary BAs into secondary BAs through deconjugation and dehydroxylation reactions, largely involving *Lactobacillus*, *Bacteroides*, *Enterococcus* and *Clostridium* spp. [164,177]. BA signaling through Farnesoid X-activated receptors (FXR) and G-protein-coupled bile acid receptors (TGR5) modulates cholesterol biosynthesis and inflammatory pathways relevant to CVD [177,187]. FXR activation regulates lipid metabolism and flavin-containing monooxygenase 3 (FMO3), a key enzyme in TMAO synthesis, whereas TGR5 activation exerts anti-inflammatory and anti-atherogenic effects via nuclear factor kappa-light-chain-enhancer of activated B cells (NF-κB) inhibition [188]. In contrast, pregnane X receptor (PXR) signaling may enhance macrophage CD36 expression and lipid uptake, promoting plaque growth [189]. Emerging data point to altered bile acid profiles and gut microbiota-derived LPS/leaky gut endotoxaemia as additional mechanisms linking microbial ecology to vascular inflammation and cholesterol metabolism [177].
- Emerging evidence suggests that, in addition to hepatic bile acid synthesis, the gut microbiota actively participates in cholesterol metabolism by converting dietary or endogenous cholesterol into coprostanol, a sterol that is poorly absorbed and therefore eliminated in feces [177,190]. This transformation is primarily mediated by bacterial species from the *Eubacterium* and *Bacteroides* genera, such as *E. coprostanoligenes* and *Bacteroides* strain D8, although other microbial contributors are likely yet to be identified [190]. Experimental studies in animal models have shown that supplementation with coprostanol-producing bacteria can substantially lower plasma cholesterol concentrations, with effects persisting for several weeks after treatment discontinuation [191]. In contrast, human investigations remain inconclusive due to small study populations, limited microbial isolation success and demographic variability [192]. Moreover, the specific microbial genes and enzymes driving intestinal cholesterol conversion are not fully defined, emphasizing the need for further mechanistic studies to clarify the role of these pathways in cholesterol regulation and cardiovascular protection [192].
- Additional microbial metabolites, including succinate, imidazole propionate (ImP) and tryptophan (Trp) derivatives, also modulate vascular inflammation. Succinate acts as a pro-inflammatory ligand for SUCNR1/GPR91, stimulating HIF-1α, IL-1β and ROS generation [177,193]. Elevated serum succinate correlates with coronary inflammation and enhanced NLRP3 inflammasome activation, linking metabolic stress to vascular damage [177,194]. Similarly, ImP, produced from histidine by specific *Clostridioides* species, interferes with insulin and AMPK signaling, promoting endothelial dysfunction and metabolic inflammation [179,195]. Finally, Trp metabolism yields multiple bioactive compounds, such as indole-3-propionic acid (IPA) and kynurenine pathway intermediates, that engage aryl hydrocarbon receptor (AhR) signaling to regulate macrophage activity and immune tolerance [196,197,198,199]. Reduced IPA and *Peptostreptococcus*-mediated Trp conversion correlate with impaired ABCA1/miR-142-5p signaling, increased foam-cell formation and aggravated atherosclerosis [199].

### 4.3. Microbial Taxa of Interest

- Gut microbiome: An elevated Firmicutes/Bacteroidetes ratio, indicative of dysbiosis, has been recurrently observed in patients with atherosclerotic disease and associated metabolic comorbidities [159]. Taxa in the Enterobacteriaceae family (e.g., *Escherichia coli*) are enriched in atherosclerosis and correlate with pro-inflammatory gene expression [200]. *Veillonella* and *Streptococcus* spp., which appear enriched in plaque samples and peripheral circulation, are emerging as potential biomarkers of atherogenic microbiota [201,202,203].
- Oral microbiome: Periodontal pathogens including *Porphyromonas gingivalis* and *Aggregatibacter actinomycetemcomitans* have been linked to vascular inflammation and atherosclerosis through mechanisms such as molecular mimicry, direct invasion of vascular tissues and induction of systemic inflammatory responses [40,204,205,206,207].

Several fundamental questions remain at the forefront of cardiovascular microbiome research. A primary objective is to determine whether distinct microbial and metabolomic signatures possess sufficient specificity and reproducibility to serve as prognostic or diagnostic biomarkers for atherosclerotic disease [161].

Equally important is the rigorous evaluation of microbiota-modulating interventions, including dietary modification, administration of prebiotics or probiotics and selective inhibition of microbial enzymatic pathways, with regard to their translational applicability, safety and efficacy in human populations [208].

Moreover, the intricate interdependence between host determinants such as genetic architecture, metabolic phenotype, environmental exposures and pharmacologic treatments and their collective influence on the gut–vascular axis, remains insufficiently elucidated. Addressing these unresolved issues will require comprehensive, longitudinal and multicentric investigations integrating advanced multi-omic profiling and high-resolution vascular imaging to delineate causal mechanisms and identify clinically actionable microbial targets for precision cardiovascular medicine [209].

## 5. Shared Mechanisms Linking Allergic Asthma and Atherosclerosis via Microbiota

### 5.1. Dysbiosis as a Common Inflammatory Driver

**Figure 2:**
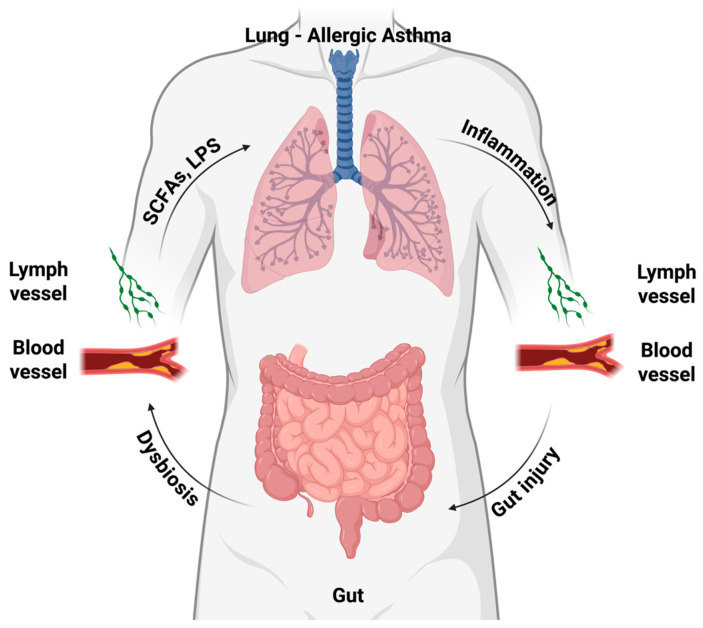
The gut–lung–vascular axis in allergic asthma and atherosclerosis. This figure illustrates how alterations in the gut microbiota influence immune and inflammatory activity across the intestines, lungs and vasculature. Under healthy conditions, a balanced microbial community maintains epithelial barrier integrity and supports immune tolerance. When dysbiosis develop (whether caused by diet, medications or other factors), the gut barrier becomes disrupted, permitting microbial metabolites and endotoxins to enter the circulation. These circulating signals activate systemic inflammatory pathways, including toll-like receptor- and NF-κB-mediated responses, resulting in persistent low-grade inflammation. The resulting immune activation can intensify airway hyperresponsiveness and allergic inflammation in asthma, while also contributing to endothelial dysfunction, lipid oxidation and plaque development in atherosclerosis. Continuous exchange of cytokines, immune cells and microbiota-derived products between the gut, lungs and vessels forms the gut–lung–vascular axis, emphasizing the shared immunometabolic mechanisms linking respiratory and cardiovascular disease. Created in BioRender. Zimbru, E. (2025) https://BioRender.com/5qfwvi1.

**Figure 3:**
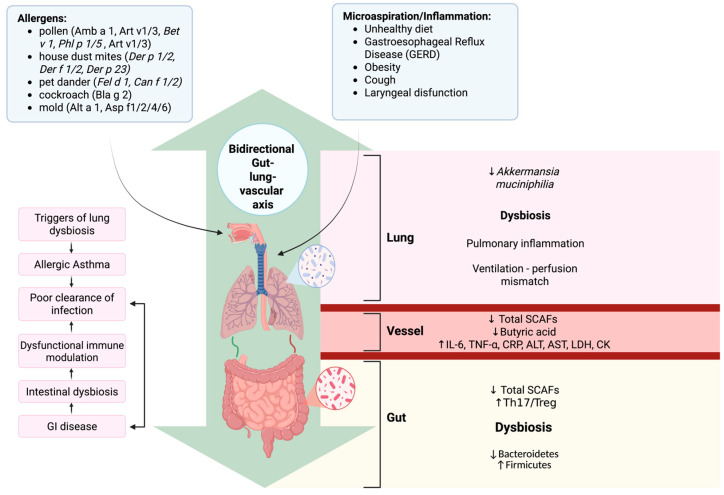
The bidirectional gut–lung–vascular axis linking dysbiosis, inflammation and immune dysfunction in allergic asthma and atherosclerosis. This figure illustrates the interconnected mechanisms through which alterations in the gut microbiota (intestinal dysbiosis) contribute to pulmonary and vascular pathology. Gastrointestinal disease and gut dysbiosis can impair immune modulation, leading to systemic inflammation, poor clearance of infections and the development or exacerbation of allergic asthma. Conversely, lung dysbiosis and pulmonary inflammation, driven by allergens such as pollen, house dust mites, pet dander, mold or cockroach antigens, can further disrupt immune homeostasis and influence gut microbial balance through systemic inflammatory signaling. Microaspiration and inflammatory conditions, including gastroesophageal reflux disease (GERD), obesity and laryngeal dysfunction, aggravate both gut and lung inflammation. Within the gut–lung–vascular axis, dysbiosis results in decreased production of short-chain fatty acids (SCFAs), particularly butyrate or propionate and increased circulating inflammatory mediators that promote endothelial dysfunction and atherogenesis. This bidirectional communication between the gut, lungs and vascular system proposes a shared immunometabolic network that links asthma and atherosclerosis through chronic inflammation and barrier dysfunction. Upward arrows (↑) indicate increased levels, whereas downward arrows (↓) indicate decreased levels of the indicated parameters. SCFAs, short-chain fatty acids; IL-6, interleukin-6; TNF-α, tumor necrosis factor-α; CRP, C-reactive protein; ALT, alanine aminotransferase; AST, aspartate aminotransferase; LDH, lactate dehydrogenase; CK, creatine kinase; Th17, T helper 17 cells; Treg, regulatory T cells. Created in BioRender. Zimbru, E. (2025) https://BioRender.com/e8zt5fd.

Recent studies indicate that allergic asthma and atherosclerosis, traditionally viewed as separate airway and vascular disorders, share microbiome-driven, immunometabolic and microRNA-mediated pathways along a gut–lung–vascular axis (Figure 2 and Figure 3, Table 1, Table 2 and Table 3) [4,138,161].

The Burkitt hypothesis, first proposed in the 1970s and later revisited in recent decades, postulated that the low incidence of “Western diseases” such as colon cancer, cardiovascular and inflammatory/metabolic disorders among rural African populations was attributable to their high dietary fiber intake [210,211,212]. Contemporary epidemiological and mechanistic studies have confirmed that fiber deficiency is strongly associated with increased risks of asthma and also atherosclerosis, largely through gut microbiota dysbiosis and reduced production of SCFAs, key metabolites with anti-inflammatory, immunomodulatory and barrier-protective properties [31,65,136,213]. Conversely, sustained consumption of fiber-rich diets enhances microbial fermentation, restores SCFA levels and supports systemic metabolic and immune homeostasis. Data suggest that increasing dietary fiber intakes by 15 g per day or to 35 g per day could extend lifespan, improve cardiometabolic health and substantially reduce long-term healthcare costs [214,215].

In both conditions, intestinal dysbiosis (reduced diversity and loss of beneficial commensals) associates with disease onset/severity in longitudinal and cross-sectional human studies, including pediatric cohorts for asthma and cardiometabolic cohorts for vascular disease [216,217,218].

### 5.2. Microbial Metabolites Bridging Lung and Vascular Physiopathology

A recurring signature of dysbiosis is reduced SCFA tone due to depletion of SCFA-producing taxa (e.g., *Faecalibacterium*, *Roseburia*) and low fiber intake. SCFAs promote epithelial barrier integrity, induce Treg differentiation through GPCR signaling and HDAC inhibition and dampen type-2/ILC2 and Th17-skewed airway inflammation; human cohorts link higher early-life or maternal SCFAs with lower risk of atopy/asthma [219,220]. Loss of barrier function increases translocation of microbe-derived products (metabolic endotoxemia), including LPS, which activates TLR-NF-κB signaling in distal tissues. In the airway, this fuels Th2/Th17 inflammation and bronchial hyperresponsiveness; in the vasculature, LPS/TLR signaling drives endothelial dysfunction, macrophage activation and foam-cell formation, which are central processes in atherogenesis [221,222].

Microbial metabolites also bridge gut and vessel biology. TMAO, generated from dietary choline/carnitine by gut microbes, is associated with adverse cardiovascular events in meta-analyses and cohort studies. It is also mechanistically linked to impaired cholesterol metabolism and increased vascular inflammation [223].

Beyond SCFAs and TMAO, BA signaling has emerged as a shared regulator. Microbiota-modified secondary BAs signal via FXR and the GPCR TGR5 on immune and barrier cells to restrain NF-κB-driven inflammation and remodel macrophage polarization; experimental and translational work suggests BA-receptor pathways influence pulmonary immunity and vascular tone, providing another conduit between gut metabolism and airway/vascular inflammation [220,224].

### 5.3. Convergent Immune and Cytokine Pathways

A convergent pro-inflammatory cytokine environment is increasingly recognized as a mechanistic bridge between airway and vascular pathology. Elevated levels of IL-6, IL-1β, IL-17, TNF-α and TGF-β orchestrate chronic immune activation and tissue remodeling within both pulmonary and vascular compartments. Through the activation of transcriptional regulators such as NF-κB and JAK/STAT, these cytokines amplify downstream inflammatory signaling, induce adhesion molecule expression and promote fibroblast and smooth muscle proliferation. The persistence of this signaling milieu favors structural changes in the bronchial wall and vascular endothelium, sustaining airway hyperresponsiveness and vascular dysfunction [225,226].

In both conditions, innate lymphoid cells (ILC2 and ILC3) and macrophage polarization toward pro-inflammatory M1 phenotypes amplify these cytokine cascades, perpetuating tissue infiltration and impaired resolution. The resulting oxidative stress, driven by NADPH oxidase, myeloperoxidase and mitochondrial ROS, induces endothelial activation and epithelial barrier dysfunction, key pathogenic events that contribute to both airway hyperresponsiveness and endothelial atherogenesis [227,228].

### 5.4. Microbial Signatures Across Respiratory and Vascular Systems

Respiratory ecosystems mirror these systemic links: enrichment of pathobiont-leaning genera such as *Moraxella*, *Haemophilus* and *Streptococcus* is repeatedly observed in airway microbiomes of children with asthma and relates to exacerbation-prone phenotypes, indicating that microbial community structure can amplify airway inflammation once systemic pro-inflammatory cues are present [229].

Oral–gut translocation may also contribute to vascular disease. DNA from oral taxa such as *Streptococcus*, *Veillonella* and *Porphyromonas gingivalis* has been detected in human atherosclerotic plaques, experimental studies show that *Porphyromonas gingivalis* can aggravate plaque biology via TLR-dependent effects on vascular cells and defective efferocytosis [201,205,206,230].

### 5.5. Integrative Concept: The Gut–Lung–Vascular Axis

Together, these observations support a unifying model: dysbiosis reduces SCFA-mediated immune tolerance and barrier protection, increases systemic exposure to microbial products (e.g., LPS) and pro-atherogenic metabolites (e.g., TMAO) and perturbs BA signaling, changes that converge on shared inflammatory nodes (TLR/NF-κB, Th2/Th17 programs) in the lungs and vessel walls [166,221,222]. Clinically, the confluence of these biological pathways is evident in the coexistence of asthma and atherosclerosis [7]. Individuals with asthma exhibit an increased burden of cardiovascular outcomes, including myocardial infarction, cerebrovascular events and markers of subclinical atherosclerosis, when compared with non-asthmatic populations [3,5,7,231]. Moreover, patients with inadequately controlled asthma frequently display a clustering of cardiometabolic disturbances, such as abdominal obesity, insulin resistance, atherogenic lipid profiles and persistent low-grade inflammation, which collectively exacerbate disease severity and compromise therapeutic responsiveness [2,35,228,232,233].

This pattern of multimorbidity reinforces the idea that the gut–lung–vascular axis provides an integrated explanatory model. In this context, disturbances in the gut microbiota are not limited to gastrointestinal or metabolic consequences, but can simultaneously influence airway inflammation and vascular pathology [177,203,218]. In this way, immune and metabolic signals derived from the microbiota can shape the evolution of respiratory and cardiovascular function in the same individual, providing a unifying mechanism behind the co-expression of asthma and ASCVD.

According to a recent article, a high-fructose diet aggravates metabolic, inflammatory and vascular dysfunction in allergic asthma, while rosuvastatin ameliorates dyslipidemia, systemic inflammation, endothelial reactivity and airway remodeling [234].

The microbiome provides interface modulating these immune and redox processes. Gut dysbiosis, characterized by the loss of *Faecalibacterium prausnitzii*, *Roseburia* and *Bacteroides* species, reduces SCFAs production, weakening Treg induction and enabling unchecked Th17/ILC3 activation [31,166,172,220,235]. Meanwhile, microbial metabolites such as TMAO and LPS trigger endothelial and macrophage TLR signaling, bridging intestinal dysbiosis to systemic vascular inflammation [24,161]. In parallel, airway microbiota alterations, including enrichment of *Haemophilus*, *Moraxella* and *Streptococcus* mirror inflammatory signatures of vascular dysbiosis, suggesting microbial–immune crosstalk between airway and cardiovascular compartments [43,144,164,165].

Complementary to the gut–lung–vascular interactions discussed in our manuscript, recent work on dermatological conditions also highlights the systemic impact of intestinal dysbiosis. A review on chronic spontaneous urticaria reports that reduced microbial diversity, depletion of SCFA-producing bacteria and expansion of Proteobacteria may contribute to increased intestinal permeability, systemic immune activation and mast cell dysregulation, emphasizing the broader relevance of microbiota-driven immune disturbances across organ systems [236].

**Table 1:** Key microbial taxa and metabolites in allergic asthma and atherosclerosis.

Condition	Key Microbial Taxa	Key Metabolites	Functional Role	References
Allergic Asthma	*Haemophilus* *Moraxella* *Streptococcus*	-	Enriched in airway microbiomes of children with asthma; associated with wheezing/exacerbations and disease severity.	[229]
	-	SCFAs (butyrate, acetate, propionate)	Linked with protection against allergic disease and dampen airway inflammation via Tregs/MDSCs.	[31,146]
	Clostridia Bacteroidetes	SCFAs	Early-life depletion of SCFA-producing taxa is associated with atopy/asthma; restoration is protective.	[31]
	*Bifidobacterium* *Lachnospira* *Veillonella* *Faecalibacterium*	SCFAs lactate	Lower early-life abundance of these gut commensals predicts increased risk of wheezing and childhood asthma; their presence supports epithelial barrier integrity and tolerogenic immune programming.	[237]
	-	Tryptophan metabolites (indole-3-carbinol, indole-3-acetic acid, kynurenine)	Activate AhR pathways to reduce airway inflammation and improve asthma outcomes.	[238]
	-	Secondary bile acids	Bile acid-FXR/TGR5 signaling modulates lung immunity and suppresses type-2 inflammation.	[28]
Atherosclerosis	Firmicutes Bacteroidetes	SCFAs	SCFAs exert anti-inflammatory and vascular-protective effects; reduced SCFA tone linked to CVD risk.	[239]
	Enterobacteriaceae	TMAO	Microbial conversion of choline/carnitine to TMAO promotes atherosclerosis and adverse cardiovascular events.	[240]
	*Streptococcus* *Veilonella* *Porphyromonas gingivalis*	LPS	Oral taxa present in plaques; endotoxin signaling (TLR4) drives vascular inflammation and lesion progression.	[201,230]
	Enterobacteriaceae Clostridia	Secondary bile acids	Microbiota-modified bile acids regulate lipid metabolism and vascular inflammation via FXR/TGR5 pathways.	[239]
	Aromatic amino acid–metabolizing gut consortia	Phenylacetylglutamine (PAGln)	PAGln, derived from microbial phenylalanine metabolism, enhances platelet reactivity through adrenergic receptors and is associated with higher risk of major adverse cardiovascular events.	[241,242]

**Table 2:** Integrative evidence from human and experimental studies demonstrating gut–lung axis involvement in asthma development and progression.

Study (Year)	Model/ Population	Main Findings	Key Microbial/ Metabolic Changes	Proposed Mechanisms/ Outcomes
Kim YC et al. (2024) [32]	Human cohort review	Provides overview of clinical and experimental evidence demonstrating that gut microbial imbalance correlates with asthma severity and exacerbations.	Depletion of SCFA-producers (*Faecalibacterium, Roseburia*); lower fecal SCFAs.	↓ SCFAs → impaired Treg induction, enhancedTh2 inflammation and airway hyperresponsiveness.
Boulund U. et al. (2025) [93]	Review (focus on early-life cohorts)	Microbial exposures during infancy and early gut colonization patterns are strong determinants of subsequent asthma susceptibility	↓ *Bifidobacterium* and other early colonizers; disrupted microbial succession during the first months of life.	Early dysbiosis interferes with immune maturation → higher allergic sensitization and increased risk of childhood asthma.
Zheng XW et al. (2024) [243]	Human cohort/genetics integration (Mendelian randomization)	Identified genetic links between gut microbial traits and asthma risk; complex taxa associations (some complex and sometimes inconsistent associations across taxa).	Context-dependent patterns, including reduced abundance of certain SCFA-producing genera but variable signals for taxa (e.g., *Roseburia*).	Indicates that host–microbe interactions in asthma are multifactorial and cannot be reduced to simple, linear taxon-to-effect relationships.
Ramar et al. (2025) [244]	Mechanistic humanized/cell-report studies	Provide emerging causal evidence that defined gut microbial communities can shape susceptibility to asthma, particularly when hosts are exposed to environmental particulate triggers.	Specific live bacterial consortia were shown to modulate airway inflammatory responses in colonized hosts.	Supports causality: gut microbes can prime lung immunity through epigenetic pathways, such as DNA methylation changes in dendritic cells, thereby driving asthma-like phenotypes.

**Table 3:** Integrative evidence from human and experimental studies demonstrating gut–vascular axis involvement in atherosclerosis development and progression.

Study (Year)	Model/ Population	Main Findings	Key Microbial/ Metabolic Changes	Proposed Mechanisms/ Outcomes
Zhu Y. et al. (2020) [245]	Review integrating experimental and preclinical evidence	Proposed central role of TMA/TMAO pathway and other metabolites in promoting endothelial dysfunction and atherogenesis.	↑ TMAO levels associated with increased activity of TMA-producing taxa such as Clostridia and *Desulfovibrio*, together with disruptions in BA metabolism.	TMAO enhances foam cell formation, promotes vascular inflammation and contributes to pro-thrombotic remodeling of the vessel wall.
Li X. et al. (2021) [246]	ApoE−/− mouse model	Antibiotic treatment or targeted modulation of the gut microbiota can alter atherosclerotic plaque burden by influencing TMA/TMAO production.	↓ TMA-generating bacterial taxa accompanied by decreased circulating TMAO levels.	Lower TMAO limits macrophage foam cell formation and vascular inflammatory pathways → smaller atherosclerotic lesions.
Pala B. et al. (2024) [247]	Human carotid intima–media thickness (IMT) cohort	Gut microbiome composition correlated with carotid IMT and plaque characteristics, linking microbial patterns to early vascular remodeling.	↑ *Prevotella* spp., altered Bacteroidetes/Firmicutes ratios; ↑ LPS-associated microbial pathways.	Microbially derived LPS and related metabolites correlate with endothelial dysfunction and markers of subclinical atherosclerosis.
Mao Y. et al. (2024) [165]	Review/multi-study synthesis	Integrative overview of how gut microbial alterations contribute to different stages of atherosclerosis, emphasizing the role of key microbial metabolites (TMAO, SCFAs, BA).	↓ SCFAs (anti-inflammatory), ↑ TMAO and specific BA derivatives in patients with increased cardiovascular risk.	Metabolite shifts modulate vascular inflammation, lipid metabolism and immune cell recruitment, thereby shaping atherosclerotic disease progression.
Jarmukhanov Z. et al. (2024) [248]	Systematic review/meta-analysis	↑ TMAO consistently associated with higher CVD and atherosclerosis risk across multiple human cohorts.	↑ circulating TMAO across cohorts; microbial taxa linked to TMA production (e.g., *Escherichia/Shigella*, *Klebsiella*).	Supports the concept of a microbe–metabolite–vascular risk pathway, with TMAO emerging as a prognostic biomarker for adverse cardiovascular outcomes.
Zhou Y. et al. (2024) [249]	Mechanistic review/multi-omics	Multi-omics evidence links gut microbial metabolome (TMAO, BA) to vascular NF-κB and inflammasome activation.	↑ TMAO and pro-inflammatory BA; ↓ protective SCFAs.	Activation of endothelial inflammatory signaling (NF-κB, NLRP3) → plaque development, progression and destabilization.

## 6. Future Directions

Future research should aim to deepen our understanding of the intricate interplay between gut microbial communities and host immune responses along the gut–lung–vascular axis. Integrative multi-omics approaches, combining metagenomics, metabolomics, transcriptomics and proteomics, are essential for comprehensively mapping the dynamic interactions between the microbiota and host pathways in both asthma and atherosclerosis. For instance, studies of the gut–lung axis have already demonstrated that gut-derived metabolites and immune signatures modulate distant organ immunity, including pulmonary responses [27,83,250]. Likewise, in the context of cardiovascular disease, a growing body of evidence supports a gut–vascular axis, where microbial dysbiosis and microbial-derived metabolites such as TMAO and SCFAs influence endothelial function, inflammation and atherogenesis [159,160,165].

Furthermore, investigation into the gut–lung–vascular axis may elucidate shared mechanistic pathways and identify key microbial or metabolite mediators that link respiratory and cardiovascular pathology. Uncovering the mediators of cross-organ signaling (for example, gut-derived immune cells or metabolites migrating via the circulation or lymphatics) remains an important gap in the current knowledge.

Clinically, a major priority is validating specific microbial and metabolite signatures as biomarkers to identify individuals at increased respiratory and cardiovascular risk and to enhance current risk prediction models. Longitudinal studies combining microbiome profiling with clinical outcomes could enable better stratification of asthmatic patients at higher cardiovascular risk and cardiovascular patients prone to respiratory complications.

Therapeutically, microbiota-targeted interventions offer promising adjuncts to standard care. These include dietary strategies (high-fiber and fermented foods), carefully selected probiotic or synbiotic formulations, pharmacologic inhibition of microbial pathways involved in harmful metabolite production (such as TMA), and, in selected cases, fecal microbiota transplantation. In asthma, such approaches may help restore immune tolerance and reduce exacerbations, while in ASCVD they may improve lipid metabolism, endothelial function and vascular inflammation. Ultimately, integrating multi-omics technologies will be critical for matching patients to the most effective microbiome-based interventions, enabling personalized treatment strategies rather than uniform supplementation approaches.

Advancement of personalized, microbiome-targeted therapies, specific to individual microbial profiles and immune signatures, holds considerable promise for optimizing clinical outcomes and ameliorating disease progression. Recent reviews emphasize that precision microbiome interventions (e.g., probiotics, prebiotics, fecal microbiota transplantation) may become feasible, but their success will depend on the integration of multi-omics datasets and immune phenotyping [34,140,151,251].

Ultimately, combining these results may enable the development of preventive strategies, including dietary, lifestyle or microbial interventions, aimed at restoring microbial-host homeostasis and reducing systemic inflammation before the disease progresses to a clinically evident stage.

## 7. Conclusions

Current evidence shows that gut microbiota plays a unifying role in linking allergic asthma with atherosclerosis by influencing systemic inflammation, immune signaling and host metabolic pathways across the gut–lung–vascular axis. Distinct microbial patterns and metabolite profiles, such as SCFAs, TMAO and LPS, affect both airway and vascular function, revealing biologically convergent trajectories of disease development. These findings open new translational opportunities: microbiome-informed diagnostics, personalized therapeutic approaches and interventions that modulate microbial metabolites may offer innovative ways to prevent or ameliorate these coexisting disorders. Incorporating microbiome evaluation into clinical risk assessment and therapeutic planning could ultimately enhance patient management, creating a more integrated and personalized framework for individuals with overlapping respiratory and cardiovascular diseases.

## Data Availability

No new data were created or analyzed in this study. Data sharing is not applicable to this article.

## References

[1] Wecker H., Tizek L., Ziehfreund S., Kain A., Traidl-Hoffmann C., Zimmermann G.S., Scala E., Elberling J., Doll A., Boffa M.J. (2023). Impact of Asthma in Europe: A Comparison of Web Search Data in 21 European Countries. World Allergy Organ. J..

[2] Aggarwal K., Bansal V., Mahmood R., Kanagala S.G., Jain R. (2025). Asthma and Cardiovascular Diseases: Uncovering Common Ground in Risk Factors and Pathogenesis. Cardiol. Rev..

[3] Hirata T. (2023). Asthma as Risk for Incident Cardiovascular Disease and Its Subtypes. Hypertens. Res..

[4] Zimbru R.-I., Zimbru E.-L., Bojin F.-M., Haidar L., Andor M., Harich O.O., Tănasie G., Tatu C., Mailat D.-E., Zbîrcea I.-M. (2025). Connecting the Dots: How MicroRNAs Link Asthma and Atherosclerosis. Int. J. Mol. Sci..

[5] Tattersall M.C., Guo M., Korcarz C.E., Gepner A.D., Kaufman J.D., Liu K.J., Barr R.G., Donohue K.M., McClelland R.L., Delaney J.A. (2015). Asthma Predicts Cardiovascular Disease Events: The Multi-Ethnic Study of Atherosclerosis. Arterioscler. Thromb. Vasc. Biol..

[6] Cazzola M., Hanania N.A., Rogliani P., Matera M.G. (2023). Cardiovascular Disease in Asthma Patients: From Mechanisms to Therapeutic Implications. Kardiol. Pol..

[7] Jiang Y., Huang X., Yu D., Xu C., Wang Y., Wang X., Shen Y. (2025). Asthma and the Risk of Cardiovascular Diseases and Mortality: A Meta-Analysis of Cohort Studies. Ther. Adv. Respir. Dis..

[8] Zhang B., Li Z.-F., An Z.-Y., Zhang L., Wang J.-Y., Hao M.-D., Jin Y.-J., Li D., Song A.-J., Ren Q. (2022). Association Between Asthma and All-Cause Mortality and Cardiovascular Disease Morbidity and Mortality: A Meta-Analysis of Cohort Studies. Front. Cardiovasc. Med..

[9] Al Samarraie A., Pichette M., Rousseau G. (2023). Role of the Gut Microbiome in the Development of Atherosclerotic Cardiovascular Disease. Int. J. Mol. Sci..

[10] Lin X., Yu Z., Liu Y., Li C., Hu H., Hu J., Liu M., Yang Q., Gu P., Li J. (2025). Gut–X Axis. iMeta.

[11] Rooks M.G., Garrett W.S. (2016). Gut Microbiota, Metabolites and Host Immunity. Nat. Rev. Immunol..

[12] Cryan J.F., O’Riordan K.J., Cowan C.S.M., Sandhu K.V., Bastiaanssen T.F.S., Boehme M., Codagnone M.G., Cussotto S., Fulling C., Golubeva A.V. (2019). The Microbiota-Gut-Brain Axis. Physiol. Rev..

[13] Goodrich J.K., Waters J.L., Poole A.C., Sutter J.L., Koren O., Blekhman R., Beaumont M., Van Treuren W., Knight R., Bell J.T. (2014). Human Genetics Shape the Gut Microbiome. Cell.

[14] Cheng C.K., Ye L., Wang Y., Wang Y.-L., Xia Y., Wong S.H.-S., Chen S., Huang Y. (2025). Exercised Gut Microbiota Improves Vascular and Metabolic Abnormalities in Sedentary Diabetic Mice through Gut-vascular Connection. J. Sport Health Sci..

[15] Zhang D., He X., Shi Y., Chen X., Yu K., Wang S. (2025). Gut Microbiota Regulate Atherosclerosis via the Gut-Vascular Axis: A Scoping Review of Mechanisms and Therapeutic Interventions. Front. Microbiol..

[16] Alexandrescu L., Suceveanu A.P., Stanigut A.M., Tofolean D.E., Axelerad A.D., Iordache I.E., Herlo A., Nelson Twakor A., Nicoara A.D., Tocia C. (2024). Intestinal Insights: The Gut Microbiome’s Role in Atherosclerotic Disease: A Narrative Review. Microorganisms.

[17] Martínez J.E., Vargas A., Pérez-Sánchez T., Encío I.J., Cabello-Olmo M., Barajas M. (2021). Human Microbiota Network: Unveiling Potential Crosstalk between the Different Microbiota Ecosystems and Their Role in Health and Disease. Nutrients.

[18] Budden K.F., Shukla S.D., Rehman S.F., Bowerman K.L., Keely S., Hugenholtz P., Armstrong-James D.P.H., Adcock I.M., Chotirmall S.H., Chung K.F. (2019). Functional Effects of the Microbiota in Chronic Respiratory Disease. Lancet Respir. Med..

[19] Liu Y., Dai J., Zhou G., Chen R., Bai C., Shi F. (2025). Innovative Therapeutic Strategies for Asthma: The Role of Gut Microbiome in Airway Immunity. J. Asthma Allergy.

[20] Ke H., Yao H., Wei P. (2025). Advances in Research on Gut Microbiota and Allergic Diseases in Children. Curr. Res. Microb. Sci..

[21] Colucci R., Moretti S. (2021). Implication of Human Bacterial Gut Microbiota on Immune-Mediated and Autoimmune Dermatological Diseases and Their Comorbidities: A Narrative Review. Dermatol. Ther..

[22] Li M., Wang F. (2021). Role of Intestinal Microbiota on Gut Homeostasis and Rheumatoid Arthritis. J. Immunol. Res..

[23] Chang C., Yuan X., Zhang X., Chen X., Li K. (2022). Gastrointestinal Microbiome and Multiple Health Outcomes: Umbrella Review. Nutrients.

[24] Muttiah B., Hanafiah A. (2025). Gut Microbiota and Cardiovascular Diseases: Unraveling the Role of Dysbiosis and Microbial Metabolites. Int. J. Mol. Sci..

[25] Nogal A., Valdes A.M., Menni C. (2021). The Role of Short-Chain Fatty Acids in the Interplay between Gut Microbiota and Diet in Cardio-Metabolic Health. Gut Microbes.

[26] Yu B., Pei C., Peng W., Zheng Y., Fu Y., Wang X., Wang W., Wang Z., Chen Y., Wang Q. (2025). Microbiota-Derived Butyrate Alleviates Asthma via Inhibiting Tfh13-Mediated IgE Production. Signal Transduct. Target. Ther..

[27] Druszczynska M., Sadowska B., Kulesza J., Gąsienica-Gliwa N., Kulesza E., Fol M. (2024). The Intriguing Connection Between the Gut and Lung Microbiomes. Pathogens.

[28] Liu Y., Zhou Y., Zhang H., Zhao K., Yang D. (2025). Gut-Lung Axis Mediates Asthma Pathogenesis: Roles of Dietary Patterns and Their Impact on the Gut Microbiota. Exp. Mol. Pathol..

[29] Suslov A.V., Panas A., Sinelnikov M.Y., Maslennikov R.V., Trishina A.S., Zharikova T.S., Zharova N.V., Kalinin D.V., Pontes-Silva A., Zharikov Y.O. (2024). Applied Physiology: Gut Microbiota and Antimicrobial Therapy. Eur. J. Appl. Physiol..

[30] Ney L.-M., Wipplinger M., Grossmann M., Engert N., Wegner V.D., Mosig A.S. (2023). Short Chain Fatty Acids: Key Regulators of the Local and Systemic Immune Response in Inflammatory Diseases and Infections. Open Biol..

[31] Sasaki M., Suaini N.H.A., Afghani J., Heye K.N., O’Mahony L., Venter C., Lauener R., Frei R., Roduit C. (2024). Systematic Review of the Association between Short-chain Fatty Acids and Allergic Diseases. Allergy.

[32] Kim Y.-C., Sohn K.-H., Kang H.-R. (2024). Gut Microbiota Dysbiosis and Its Impact on Asthma and Other Lung Diseases: Potential Therapeutic Approaches. Korean J. Intern. Med..

[33] Alagiakrishnan K., Morgadinho J., Halverson T. (2024). Approach to the Diagnosis and Management of Dysbiosis. Front. Nutr..

[34] Kleniewska P., Pawliczak R. (2024). The Link Between Dysbiosis, Inflammation, Oxidative Stress, and Asthma—The Role of Probiotics, Prebiotics, and Antioxidants. Nutrients.

[35] Tashiro H., Kuwahara Y., Takahashi K. (2025). Gut–Lung Axis in Asthma and Obesity: Role of the Gut Microbiome. Front. Allergy.

[36] Thursby E., Juge N. (2017). Introduction to the Human Gut Microbiota. Biochem. J..

[37] Pascale A., Marchesi N., Marelli C., Coppola A., Luzi L., Govoni S., Giustina A., Gazzaruso C. (2018). Microbiota and Metabolic Diseases. Endocrine.

[38] Borrego-Ruiz A., Borrego J.J. (2025). Early-Life Gut Microbiome Development and Its Potential Long-Term Impact on Health Outcomes. Microbiome Res. Rep..

[39] Zhu B., Edwards D.J., Spaine K.M., Edupuganti L., Matveyev A., Serrano M.G., Buck G.A. (2024). The Association of Maternal Factors with the Neonatal Microbiota and Health. Nat. Commun..

[40] Yang L., Yang J., Zhang T., Xie X., Wu Q. (2025). Gut Microbiota: A Novel Strategy Affecting Atherosclerosis. Microbiol. Spectr..

[41] Salazar J., Angarita L., Morillo V., Navarro C., Martínez M.S., Chacín M., Torres W., Rajotia A., Rojas M., Cano C. (2020). Microbiota and Diabetes Mellitus: Role of Lipid Mediators. Nutrients.

[42] Espírito Santo C., Caseiro C., Martins M.J., Monteiro R., Brandão I. (2021). Gut Microbiota, in the Halfway between Nutrition and Lung Function. Nutrients.

[43] Durack J., Lynch S.V. (2019). The Gut Microbiome: Relationships with Disease and Opportunities for Therapy. J. Exp. Med..

[44] Malesza I.J., Malesza M., Walkowiak J., Mussin N., Walkowiak D., Aringazina R., Bartkowiak-Wieczorek J., Mądry E. (2021). High-Fat, Western-Style Diet, Systemic Inflammation, and Gut Microbiota: A Narrative Review. Cells.

[45] Caporaso J.G., Lauber C.L., Costello E.K., Berg-Lyons D., Gonzalez A., Stombaugh J., Knights D., Gajer P., Ravel J., Fierer N. (2011). Moving Pictures of the Human Microbiome. Genome Biol..

[46] Tremaroli V., Bäckhed F. (2012). Functional Interactions between the Gut Microbiota and Host Metabolism. Nature.

[47] Barber T.M., Kabisch S., Pfeiffer A.F.H., Weickert M.O. (2023). The Effects of the Mediterranean Diet on Health and Gut Microbiota. Nutrients.

[48] Strasser B., Wolters M., Weyh C., Krüger K., Ticinesi A. (2021). The Effects of Lifestyle and Diet on Gut Microbiota Composition, Inflammation and Muscle Performance in Our Aging Society. Nutrients.

[49] Van Hul M., Cani P.D., Petitfils C., De Vos W.M., Tilg H., El-Omar E.M. (2024). What Defines a Healthy Gut Microbiome?. Gut.

[50] Pant A., Maiti T.K., Mahajan D., Das B. (2023). Human Gut Microbiota and Drug Metabolism. Microb. Ecol..

[51] Gowen R., Gamal A., Di Martino L., McCormick T.S., Ghannoum M.A. (2023). Modulating the Microbiome for Crohn’s Disease Treatment. Gastroenterology.

[52] Lee J.-Y., Bays D.J., Savage H.P., Bäumler A.J. (2024). The Human Gut Microbiome in Health and Disease: Time for a New Chapter?. Infect. Immun..

[53] Colonetti T., Saggioratto M.C., Grande A.J., Colonetti L., Junior J.C.D., Ceretta L.B., Roever L., Silva F.R., Da Rosa M.I. (2023). Gut and Vaginal Microbiota in the Endometriosis: Systematic Review and Meta-Analysis. BioMed Res. Int..

[54] Soldán M., Argalášová Ľ., Hadvinová L., Galileo B., Babjaková J. (2024). The Effect of Dietary Types on Gut Microbiota Composition and Development of Non-Communicable Diseases: A Narrative Review. Nutrients.

[55] Merra G., Noce A., Marrone G., Cintoni M., Tarsitano M.G., Capacci A., De Lorenzo A. (2020). Influence of Mediterranean Diet on Human Gut Microbiota. Nutrients.

[56] Del Chierico F., Vernocchi P., Dallapiccola B., Putignani L. (2014). Mediterranean Diet and Health: Food Effects on Gut Microbiota and Disease Control. Int. J. Mol. Sci..

[57] Nagpal R., Shively C.A., Register T.C., Craft S., Yadav H. (2019). Gut Microbiome-Mediterranean Diet Interactions in Improving Host Health. F1000Research.

[58] Cani P.D., Bibiloni R., Knauf C., Waget A., Neyrinck A.M., Delzenne N.M., Burcelin R. (2008). Changes in Gut Microbiota Control Metabolic Endotoxemia-Induced Inflammation in High-Fat Diet–Induced Obesity and Diabetes in Mice. Diabetes.

[59] Fuke N., Nagata N., Suganuma H., Ota T. (2019). Regulation of Gut Microbiota and Metabolic Endotoxemia with Dietary Factors. Nutrients.

[60] Mamun M.A.A., Rakib A., Mandal M., Singh U.P. (2025). Impact of a High-Fat Diet on the Gut Microbiome: A Comprehensive Study of Microbial and Metabolite Shifts During Obesity. Cells.

[61] Tan R., Dong H., Chen Z., Jin M., Yin J., Li H., Shi D., Shao Y., Wang H., Chen T. (2021). Intestinal Microbiota Mediates High-Fructose and High-Fat Diets to Induce Chronic Intestinal Inflammation. Front. Cell. Infect. Microbiol..

[62] Tomova A., Bukovsky I., Rembert E., Yonas W., Alwarith J., Barnard N.D., Kahleova H. (2019). The Effects of Vegetarian and Vegan Diets on Gut Microbiota. Front. Nutr..

[63] Rew L., Harris M.D., Goldie J. (2022). The Ketogenic Diet: Its Impact on Human Gut Microbiota and Potential Consequent Health Outcomes: A Systematic Literature Review. Gastroenterol. Hepatol. Bed Bench.

[64] Jiang Y., Chen Y., Chen Y., Gong X., Chen Z., Zhang X. (2025). Ketogenic Diet and Gut Microbiota: Exploring New Perspectives on Cognition and Mood. Foods.

[65] Makki K., Deehan E.C., Walter J., Bäckhed F. (2018). The Impact of Dietary Fiber on Gut Microbiota in Host Health and Disease. Cell Host Microbe.

[66] Ghosh A.N., Walsh C.J., Maiden M.J., Stinear T.P., Deane A.M. (2025). Effect of Dietary Fibre on the Gastrointestinal Microbiota during Critical Illness: A Scoping Review. World J. Crit. Care Med..

[67] Fu J., Zheng Y., Gao Y., Xu W. (2022). Dietary Fiber Intake and Gut Microbiota in Human Health. Microorganisms.

[68] Wastyk H.C., Fragiadakis G.K., Perelman D., Dahan D., Merrill B.D., Yu F.B., Topf M., Gonzalez C.G., Van Treuren W., Han S. (2021). Gut-Microbiota-Targeted Diets Modulate Human Immune Status. Cell.

[69] Leeuwendaal N.K., Stanton C., O’Toole P.W., Beresford T.P. (2022). Fermented Foods, Health and the Gut Microbiome. Nutrients.

[70] Zhao Q., Chen Y., Huang W., Zhou H., Zhang W. (2023). Drug-Microbiota Interactions: An Emerging Priority for Precision Medicine. Signal Transduct. Target. Ther..

[71] Hu X., Li H., Zhao X., Zhou R., Liu H., Sun Y., Fan Y., Shi Y., Qiao S., Liu S. (2021). Multi-Omics Study Reveals That Statin Therapy Is Associated with Restoration of Gut Microbiota Homeostasis and Improvement in Outcomes in Patients with Acute Coronary Syndrome. Theranostics.

[72] She J., Sun L., Yu Y., Fan H., Li X., Zhang X., Zhuo X., Guo M., Liu J., Liu P. (2024). A Gut Feeling of Statin. Gut Microbes.

[73] Wang T., Lan Q., Deng H., Han W., Zhang R., Zhong J. (2025). Interactions between Gut Microbiota and Cardiovascular Drugs: Effects on Drug Therapeutic Effect and Side Effect. Front. Cardiovasc. Med..

[74] Zimbru R.-I., Grijincu M., Tănasie G., Zimbru E.-L., Bojin F.-M., Buzan R.-M., Tamaș T.-P., Cotarcă M.-D., Harich O.O., Pătrașcu R. (2025). Breaking Barriers: The Detrimental Effects of Combined Ragweed and House Dust Mite Allergen Extract Exposure on the Bronchial Epithelium. Appl. Sci..

[75] Mousa S., Sarfraz M., Mousa W.K. (2023). The Interplay between Gut Microbiota and Oral Medications and Its Impact on Advancing Precision Medicine. Metabolites.

[76] Peltak S.N., Steen T.Y. (2025). The Impact of Antibiotic Use on the Human Gut Microbiome: A Review. Georget. Med. Rev..

[77] Patangia D.V., Anthony Ryan C., Dempsey E., Paul Ross R., Stanton C. (2022). Impact of Antibiotics on the Human Microbiome and Consequences for Host Health. MicrobiologyOpen.

[78] Wang S., Ju D., Zeng X. (2024). Mechanisms and Clinical Implications of Human Gut Microbiota-Drug Interactions in the Precision Medicine Era. Biomedicines.

[79] Simonyte Sjödin K., Vidman L., Rydén P., West C.E. (2016). Emerging Evidence of the Role of Gut Microbiota in the Development of Allergic Diseases. Curr. Opin. Allergy Clin. Immunol..

[80] Huang E.Y., Inoue T., Leone V.A., Dalal S., Touw K., Wang Y., Musch M.W., Theriault B., Higuchi K., Donovan S. (2015). Using Corticosteroids to Reshape the Gut Microbiome: Implications for Inflammatory Bowel Diseases. Inflamm. Bowel Dis..

[81] Su X., Tian Z., Fang Y., Zhou S., Ma S. (2025). Effects of High-Dose Glucocorticoids on Gut Microbiota in the Treatment of Graves’ Ophthalmopathy. Microbiol. Spectr..

[82] Barcik W., Boutin R.C.T., Sokolowska M., Finlay B.B. (2020). The Role of Lung and Gut Microbiota in the Pathology of Asthma. Immunity.

[83] Zhang M., Qin Z., Huang C., Liang B., Zhang X., Sun W. (2024). The Gut Microbiota Modulates Airway Inflammation in Allergic Asthma through the Gut-Lung Axis Related Immune Modulation: A Review. Biomol. Biomed..

[84] Lv J., Zhang Y., Liu S., Wang R., Zhao J. (2025). Gut-Lung Axis in Allergic Asthma: Microbiota-Driven Immune Dysregulation and Therapeutic Strategies. Front. Pharmacol..

[85] Imhann F., Bonder M.J., Vich Vila A., Fu J., Mujagic Z., Vork L., Tigchelaar E.F., Jankipersadsing S.A., Cenit M.C., Harmsen H.J.M. (2016). Proton Pump Inhibitors Affect the Gut Microbiome. Gut.

[86] Zhang X., Li Q., Xia S., He Y., Liu Y., Yang J., Xiao X. (2024). Proton Pump Inhibitors and Oral–Gut Microbiota: From Mechanism to Clinical Significance. Biomedicines.

[87] Li X.-B., Chu X.-J., Cao N.-W., Wang H., Fang X.-Y., Fan Y.-G., Li B.-Z., Ye D.-Q. (2022). Proton Pump Inhibitors Induce Changes in the Gut Microbiome Composition of Systemic Lupus Erythematosus Patients. BMC Microbiol..

[88] Tuteja S., Ferguson J.F. (2019). Gut Microbiome and Response to Cardiovascular Drugs. Circ. Genom. Precis. Med..

[89] Sun C., Wang Z., Hu L., Zhang X., Chen J., Yu Z., Liu L., Wu M. (2022). Targets of Statins Intervention in LDL-C Metabolism: Gut Microbiota. Front. Cardiovasc. Med..

[90] Le Bastard Q., Berthelot L., Soulillou J.-P., Montassier E. (2021). Impact of Non-Antibiotic Drugs on the Human Intestinal Microbiome. Expert Rev. Mol. Diagn.

[91] Skillington O., Mills S., Gupta A., Mayer E.A., Gill C.I.R., Del Rio D., O’Riordan K.J., Cryan J.F., Ross R.P., Stanton C. (2021). The Contrasting Human Gut Microbiota in Early and Late Life and Implications for Host Health and Disease. Nutr. Healthy Aging.

[92] Pattaroni C., Marsland B.J., Harris N.L. (2025). Early-Life Host–Microbial Interactions and Asthma Development: A Lifelong Impact?. Immunol. Rev..

[93] Boulund U., Thorsen J., Trivedi U., Tranæs K., Jiang J., Shah S.A., Stokholm J. (2025). The Role of the Early-Life Gut Microbiome in Childhood Asthma. Gut Microbes.

[94] Bradley E., Haran J. (2024). The Human Gut Microbiome and Aging. Gut Microbes.

[95] Caldarelli M., Rio P., Marrone A., Giambra V., Gasbarrini A., Gambassi G., Cianci R. (2024). Inflammaging: The Next Challenge—Exploring the Role of Gut Microbiota, Environmental Factors, and Sex Differences. Biomedicines.

[96] Ai X., Huang C., Liu Q., Duan R., Ma X., Li L., Shu Z., Miao Y., Shen H., Lv Y. (2025). Gut Microbiome Dynamics and Functional Shifts in Healthy Aging: Insights from a Metagenomic Study. Front. Microbiol..

[97] Bian G., Gloor G.B., Gong A., Jia C., Zhang W., Hu J., Zhang H., Zhang Y., Zhou Z., Zhang J. (2017). The Gut Microbiota of Healthy Aged Chinese Is Similar to That of the Healthy Young. mSphere.

[98] DeJong E.N., Surette M.G., Bowdish D.M.E. (2020). The Gut Microbiota and Unhealthy Aging: Disentangling Cause from Consequence. Cell Host Microbe.

[99] Singh H., Torralba M.G., Moncera K.J., DiLello L., Petrini J., Nelson K.E., Pieper R. (2019). Gastro-Intestinal and Oral Microbiome Signatures Associated with Healthy Aging. GeroScience.

[100] Haran J.P., Bucci V., Dutta P., Ward D., McCormick B. (2018). The Nursing Home Elder Microbiome Stability and Associations with Age, Frailty, Nutrition and Physical Location. J. Med. Microbiol..

[101] Haran J.P., Bhattarai S.K., Foley S.E., Dutta P., Ward D.V., Bucci V., McCormick B.A. (2019). Alzheimer’s Disease Microbiome Is Associated with Dysregulation of the Anti-Inflammatory P-Glycoprotein Pathway. mBio.

[102] Kemis J.H., Linke V., Barrett K.L., Boehm F.J., Traeger L.L., Keller M.P., Rabaglia M.E., Schueler K.L., Stapleton D.S., Gatti D.M. (2019). Genetic Determinants of Gut Microbiota Composition and Bile Acid Profiles in Mice. PLoS Genet..

[103] Guzior D.V., Quinn R.A. (2021). Review: Microbial Transformations of Human Bile Acids. Microbiome.

[104] Bubier J.A., Chesler E.J., Weinstock G.M. (2021). Host Genetic Control of Gut Microbiome Composition. Mamm. Genome.

[105] Grieneisen L., Dasari M., Gould T.J., Björk J.R., Grenier J.-C., Yotova V., Jansen D., Gottel N., Gordon J.B., Learn N.H. (2021). Gut Microbiome Heritability Is Nearly Universal but Environmentally Contingent. Science.

[106] Kurilshikov A., Medina-Gomez C., Bacigalupe R., Radjabzadeh D., Wang J., Demirkan A., Le Roy C.I., Raygoza Garay J.A., Finnicum C.T., Liu X. (2021). Large-Scale Association Analyses Identify Host Factors Influencing Human Gut Microbiome Composition. Nat. Genet..

[107] Casciaro M., Di Salvo E., Pioggia G., Gangemi S. (2020). Microbiota and microRNAs in Lung Diseases: Mutual Influence and Role Insights. Eur. Rev. Med. Pharmacol. Sci..

[108] Lanaspa M., Bassat Q., Medeiros M.M., Muñoz-Almagro C. (2017). Respiratory Microbiota and Lower Respiratory Tract Disease. Expert Rev. Anti-Infect. Ther..

[109] Korpela K., De Vos W.M. (2022). Infant Gut Microbiota Restoration: State of the Art. Gut Microbes.

[110] Van Nimwegen F.A., Penders J., Stobberingh E.E., Postma D.S., Koppelman G.H., Kerkhof M., Reijmerink N.E., Dompeling E., Van Den Brandt P.A., Ferreira I. (2011). Mode and Place of Delivery, Gastrointestinal Microbiota, and Their Influence on Asthma and Atopy. J. Allergy Clin. Immunol..

[111] Edouard S., Million M., Bachar D., Dubourg G., Michelle C., Ninove L., Charrel R., Raoult D. (2018). The Nasopharyngeal Microbiota in Patients with Viral Respiratory Tract Infections Is Enriched in Bacterial Pathogens. Eur. J. Clin. Microbiol. Infect. Dis..

[112] Arrieta M.-C., Stiemsma L.T., Dimitriu P.A., Thorson L., Russell S., Yurist-Doutsch S., Kuzeljevic B., Gold M.J., Britton H.M., Lefebvre D.L. (2015). Early Infancy Microbial and Metabolic Alterations Affect Risk of Childhood Asthma. Sci. Transl. Med..

[113] Fujimura K.E., Sitarik A.R., Havstad S., Lin D.L., Levan S., Fadrosh D., Panzer A.R., LaMere B., Rackaityte E., Lukacs N.W. (2016). Neonatal Gut Microbiota Associates with Childhood Multisensitized Atopy and T Cell Differentiation. Nat. Med..

[114] Ljung A., Gio-Batta M., Hesselmar B., Imberg H., Rabe H., Nowrouzian F.L., Johansen S., Törnhage C.-J., Lindhagen G., Ceder M. (2024). Gut Microbiota Markers in Early Childhood Are Linked to Farm Living, Pets in Household and Allergy. PLoS ONE.

[115] Lehtimäki J., Thorsen J., Rasmussen M.A., Hjelmsø M., Shah S., Mortensen M.S., Trivedi U., Vestergaard G., Bønnelykke K., Chawes B.L. (2021). Urbanized Microbiota in Infants, Immune Constitution, and Later Risk of Atopic Diseases. J. Allergy Clin. Immunol..

[116] Mills M., Lee S., Piperata B.A., Garabed R., Choi B., Lee J. (2023). Household Environment and Animal Fecal Contamination Are Critical Modifiers of the Gut Microbiome and Resistome in Young Children from Rural Nicaragua. Microbiome.

[117] Huang X., Wang Z., Lei F., Liu W., Lin L., Sun T., Cao Y., Zhang X., Cai J., Li H. (2024). Association of Urban Environments with Atherosclerotic Cardiovascular Disease: A Prospective Cohort Study in the UK Biobank. Environ. Int..

[118] Brown J.R.G., Baptiste P.J., Hajmohammadi H., Nadarajah R., Gale C.P., Wu J. (2025). Impact of Neighbourhood and Environmental Factors on the Risk of Incident Cardiovascular Disease: A Systematic Review and Meta-Analysis. Eur. J. Prev. Cardiol..

[119] Rus A.-A., Mornoş C. (2022). The Impact of Meteorological Factors and Air Pollutants on Acute Coronary Syndrome. Curr. Cardiol. Rep..

[120] Ansaldo E., Farley T.K., Belkaid Y. (2021). Control of Immunity by the Microbiota. Annu. Rev. Immunol..

[121] Cianci R., Franza L., Massaro M.G., Borriello R., Tota A., Pallozzi M., De Vito F., Gambassi G. (2022). The Crosstalk between Gut Microbiota, Intestinal Immunological Niche and Visceral Adipose Tissue as a New Model for the Pathogenesis of Metabolic and Inflammatory Diseases: The Paradigm of Type 2 Diabetes Mellitus. Curr. Med. Chem..

[122] D’Amico F., Barone M., Tavella T., Rampelli S., Brigidi P., Turroni S. (2022). Host Microbiomes in Tumor Precision Medicine: How Far Are We?. Curr. Med. Chem..

[123] Piccioni A., Cicchinelli S., Valletta F., De Luca G., Longhitano Y., Candelli M., Ojetti V., Sardeo F., Navarra S., Covino M. (2022). Gut Microbiota and Autoimmune Diseases: A Charming Real World Together with Probiotics. Curr. Med. Chem..

[124] Cassini C., Zatti P.H., Angeli V.W., Branco C.S., Salvador M. (2022). Mutual Effects of Free and Nanoencapsulated Phenolic Compoundson Human Microbiota. Curr. Med. Chem..

[125] Baldanzi G., Sayols-Baixeras S., Ekblom-Bak E., Ekblom Ö., Dekkers K.F., Hammar U., Nguyen D., Ahmad S., Ericson U., Arvidsson D. (2024). Accelerometer-Based Physical Activity Is Associated with the Gut Microbiota in 8416 Individuals in SCAPIS. eBioMedicine.

[126] Aya V., Jimenez P., Muñoz E., Ramírez J.D. (2023). Effects of Exercise and Physical Activity on Gut Microbiota Composition and Function in Older Adults: A Systematic Review. BMC Geriatr..

[127] Lotti S., Dinu M., Colombini B., Amedei A., Sofi F. (2023). Circadian Rhythms, Gut Microbiota, and Diet: Possible Implications for Health. Nutr. Metab. Cardiovasc. Dis..

[128] Du Y., Li L., Gong C., Li T., Xia Y. (2022). The Diversity of the Intestinal Microbiota in Patients with Alcohol Use Disorder and Its Relationship to Alcohol Consumption and Cognition. Front. Psychiatry.

[129] Chen B., Zeng G., Sun L., Jiang C. (2024). When Smoke Meets Gut: Deciphering the Interactions between Tobacco Smoking and Gut Microbiota in Disease Development. Sci. China Life Sci..

[130] Ma L., Yan Y., Webb R.J., Li Y., Mehrabani S., Xin B., Sun X., Wang Y., Mazidi M. (2023). Psychological Stress and Gut Microbiota Composition: A Systematic Review of Human Studies. Neuropsychobiology.

[131] Zhang H., Wang Z., Wang G., Song X., Qian Y., Liao Z., Sui L., Ai L., Xia Y. (2023). Understanding the Connection between Gut Homeostasis and Psychological Stress. J. Nutr..

[132] Sudo N., Chida Y., Aiba Y., Sonoda J., Oyama N., Yu X., Kubo C., Koga Y. (2004). Postnatal Microbial Colonization Programs the Hypothalamic–Pituitary–Adrenal System for Stress Response in Mice. J. Physiol..

[133] Sudo N. (2019). Role of Gut Microbiota in Brain Function and Stress-Related Pathology. Biosci. Microbiota Food Health.

[134] Beurel E. (2024). Stress in the Microbiome-Immune Crosstalk. Gut Microbes.

[135] Tofani G.S.S., Clarke G., Cryan J.F. (2025). I “Gut” Rhythm: The Microbiota as a Modulator of the Stress Response and Circadian Rhythms. FEBS J..

[136] Ashique S., De Rubis G., Sirohi E., Mishra N., Rihan M., Garg A., Reyes R.-J., Manandhar B., Bhatt S., Jha N.K. (2022). Short Chain Fatty Acids: Fundamental Mediators of the Gut-Lung Axis and Their Involvement in Pulmonary Diseases. Chem.-Biol. Interact..

[137] Yip W., Hughes M.R., Li Y., Cait A., Hirst M., Mohn W.W., McNagny K.M. (2021). Butyrate Shapes Immune Cell Fate and Function in Allergic Asthma. Front. Immunol..

[138] Zhao X., Hu M., Zhou H., Yang Y., Shen S., You Y., Xue Z. (2023). The Role of Gut Microbiome in the Complex Relationship between Respiratory Tract Infection and Asthma. Front. Microbiol..

[139] Schenzel A., Geiger A., Nendel E., Yang Z., Krammer S., Leberle A., Brunst A.-K., Trump S., Mittler S., Rauh M. (2024). Fiber Rich Food Suppressed Airway Inflammation, GATA3 + Th2 Cells, and FcεRIα+ Eosinophils in Asthma. Front. Nutr..

[140] Kaur H., Golovko S., Golovko M.Y., Singh S., Darland D.C., Combs C.K. (2020). Effects of Probiotic Supplementation on Short Chain Fatty Acids in the *App^NL-G-F^* Mouse Model of Alzheimer’s Disease. J. Alzheimer’s Dis..

[141] Hougee S., Vriesema A.J.M., Wijering S.C., Knippels L.M.J., Folkerts G., Nijkamp F.P., Knol J., Garssen J. (2010). Oral Treatment with Probiotics Reduces Allergic Symptoms in Ovalbumin-Sensitized Mice: A Bacterial Strain Comparative Study. Int. Arch. Allergy Immunol..

[142] Angurana S.K., Bansal A., Singhi S., Aggarwal R., Jayashree M., Salaria M., Mangat N.K. (2018). Evaluation of Effect of Probiotics on Cytokine Levels in Critically Ill Children With Severe Sepsis: A Double-Blind, Placebo-Controlled Trial. Crit. Care Med..

[143] Lin E.-K., Chang W.-W., Jhong J.-H., Tsai W.-H., Chou C.-H., Wang I.-J. (2023). Lacticaseibacillus Paracasei GM-080 Ameliorates Allergic Airway Inflammation in Children with Allergic Rhinitis: From an Animal Model to a Double-Blind, Randomized, Placebo-Controlled Trial. Cells.

[144] Kim Y.J., Bunyavanich S. (2025). Microbial Influencers: The Airway Microbiome’s Role in Asthma. J. Clin. Investig..

[145] Zimbru R.-I., Zimbru E.-L., Ordodi V.-L., Bojin F.-M., Crîsnic D., Grijincu M., Mirica S.-N., Tănasie G., Georgescu M., Huțu I. (2024). The Impact of High-Fructose Diet and Co-Sensitization to House Dust Mites and Ragweed Pollen on the Modulation of Airway Reactivity and Serum Biomarkers in Rats. Int. J. Mol. Sci..

[146] Huang M.-T., Chiu C.-J., Tsai C.-Y., Lee Y.-R., Liu W.-L., Chuang H.-L., Huang M.-T. (2023). Short-Chain Fatty Acids Ameliorate Allergic Airway Inflammation via Sequential Induction of PMN-MDSCs and Treg Cells. J. Allergy Clin. Immunol. Glob..

[147] Ito T., Nakanishi Y., Shibata R., Sato N., Jinnohara T., Suzuki S., Suda W., Hattori M., Kimura I., Nakano T. (2023). The Propionate-GPR41 Axis in Infancy Protects from Subsequent Bronchial Asthma Onset. Gut Microbes.

[148] Liu X., Shao J., Liao Y.-T., Wang L.-N., Jia Y., Dong P., Liu Z., He D., Li C., Zhang X. (2023). Regulation of Short-Chain Fatty Acids in the Immune System. Front. Immunol..

[149] Wang J., Zhao Q., Zhang S., Liu J., Fan X., Han B., Hou Y., Ai X. (2025). Microbial Short Chain Fatty Acids: Effective Histone Deacetylase Inhibitors in Immune Regulation (Review). Int. J. Mol. Med.

[150] Miao Y., Zhong C., Bao S., Wei K., Wang W., Li N., Bai C., Chen W., Tang H. (2024). Impaired Tryptophan Metabolism by Type 2 Inflammation in Epithelium Worsening Asthma. iScience.

[151] Balan D., Baral T., Manu M.K., Mohapatra A.K., Miraj S.S. (2024). Efficacy of Probiotics as Adjuvant Therapy in Bronchial Asthma: A Systematic Review and Meta-Analysis. Allergy Asthma Clin. Immunol..

[152] Drago L., Cioffi L., Giuliano M., Pane M., Ciprandi G., The PROPAM Study Group (2022). A *Post Hoc* Analysis on the Effects of a Probiotic Mixture on Asthma Exacerbation Frequency in Schoolchildren. ERJ Open Res..

[153] Wen S., Yuan G., Li C., Xiong Y., Zhong X., Li X. (2022). High Cellulose Dietary Intake Relieves Asthma Inflammation through the Intestinal Microbiome in a Mouse Model. PLoS ONE.

[154] Sdona E., Ekström S., Andersson N., Håkansson N., Wolk A., Westman M., Van Hage M., Kull I., Melén E., Bergström A. (2022). Dietary Fibre in Relation to Asthma, Allergic Rhinitis and Sensitization from Childhood up to Adulthood. Clin. Transl. Allergy.

[155] Dębińska A., Sozańska B. (2022). Fermented Food in Asthma and Respiratory Allergies—Chance or Failure?. Nutrients.

[156] Lai Y., Qiu R., Zhou J., Ren L., Qu Y., Zhang G. (2025). Fecal Microbiota Transplantation Alleviates Airway Inflammation in Asthmatic Rats by Increasing the Level of Short-Chain Fatty Acids in the Intestine. Inflammation.

[157] Chakraborty P., Aravindhan V., Mukherjee S. (2023). Helminth-Derived Biomacromolecules as Therapeutic Agents for Treating Inflammatory and Infectious Diseases: What Lessons Do We Get from Recent Findings?. Int. J. Biol. Macromol..

[158] Feary J.R., Venn A.J., Mortimer K., Brown A.P., Hooi D., Falcone F.H., Pritchard D.I., Britton J.R. (2010). Experimental Hookworm Infection: A Randomized Placebo-controlled Trial in Asthma. Clin Exp. Allergy.

[159] Bui T.V.A., Hwangbo H., Lai Y., Hong S.B., Choi Y.-J., Park H.-J., Ban K. (2023). The Gut-Heart Axis: Updated Review for The Roles of Microbiome in Cardiovascular Health. Korean Circ. J..

[160] Dong Y., Xu R., Chen X., Yang C., Jiang F., Shen Y., Li Q., Fang F., Li Y., Shen X. (2023). Characterization of Gut Microbiota in Adults with Coronary Atherosclerosis. PeerJ.

[161] Tonch-Cerbu A.-K., Boicean A.-G., Stoia O.-M., Teodoru M. (2025). Gut Microbiota-Derived Metabolites in Atherosclerosis: Pathways, Biomarkers, and Targets. Int. J. Mol. Sci..

[162] Shen X., Li L., Sun Z., Zang G., Zhang L., Shao C., Wang Z. (2021). Gut Microbiota and Atherosclerosis—Focusing on the Plaque Stability. Front. Cardiovasc. Med..

[163] Witkowski M., Weeks T.L., Hazen S.L. (2020). Gut Microbiota and Cardiovascular Disease. Circ. Res..

[164] Zhang X., Gérard P. (2022). Diet-Gut Microbiota Interactions on Cardiovascular Disease. Comput. Struct. Biotechnol. J..

[165] Mao Y., Kong C., Zang T., You L., Wang L., Shen L., Ge J. (2024). Impact of the Gut Microbiome on Atherosclerosis. mLife.

[166] Du Y., He C., An Y., Huang Y., Zhang H., Fu W., Wang M., Shan Z., Xie J., Yang Y. (2024). The Role of Short Chain Fatty Acids in Inflammation and Body Health. Int. J. Mol. Sci..

[167] Zaric B.L., Radovanovic J.N., Gluvic Z., Stewart A.J., Essack M., Motwalli O., Gojobori T., Isenovic E.R. (2020). Atherosclerosis Linked to Aberrant Amino Acid Metabolism and Immunosuppressive Amino Acid Catabolizing Enzymes. Front. Immunol..

[168] Marques F.Z., Nelson E., Chu P.-Y., Horlock D., Fiedler A., Ziemann M., Tan J.K., Kuruppu S., Rajapakse N.W., El-Osta A. (2017). High-Fiber Diet and Acetate Supplementation Change the Gut Microbiota and Prevent the Development of Hypertension and Heart Failure in Hypertensive Mice. Circulation.

[169] Kasahara K., Krautkramer K.A., Org E., Romano K.A., Kerby R.L., Vivas E.I., Mehrabian M., Denu J.M., Bäckhed F., Lusis A.J. (2018). Interactions between Roseburia Intestinalis and Diet Modulate Atherogenesis in a Murine Model. Nat. Microbiol..

[170] Bartolomaeus H., Balogh A., Yakoub M., Homann S., Markó L., Höges S., Tsvetkov D., Krannich A., Wundersitz S., Avery E.G. (2019). Short-Chain Fatty Acid Propionate Protects From Hypertensive Cardiovascular Damage. Circulation.

[171] Tortelote G.G. (2025). Therapeutic Strategies for Hypertension: Exploring the Role of Microbiota-Derived Short-Chain Fatty Acids in Kidney Physiology and Development. Pediatr. Nephrol.

[172] Robles-Vera I., Toral M., De La Visitación N., Aguilera-Sánchez N., Redondo J.M., Duarte J. (2020). Protective Effects of Short-Chain Fatty Acids on Endothelial Dysfunction Induced by Angiotensin II. Front. Physiol..

[173] Xu J., Moore B.N., Pluznick J.L. (2022). Short-Chain Fatty Acid Receptors and Blood Pressure Regulation: Council on Hypertension Mid-Career Award for Research Excellence 2021. Hypertension.

[174] Ghavami A., Banpouri S., Ziaei R., Talebi S., Vajdi M., Nattagh-Eshtivani E., Barghchi H., Mohammadi H., Askari G. (2023). Effect of Soluble Fiber on Blood Pressure in Adults: A Systematic Review and Dose–Response Meta-Analysis of Randomized Controlled Trials. Nutr. J..

[175] Korsten S.G.P.J., Vromans H., Garssen J., Willemsen L.E.M. (2023). Butyrate Protects Barrier Integrity and Suppresses Immune Activation in a Caco-2/PBMC Co-Culture Model While HDAC Inhibition Mimics Butyrate in Restoring Cytokine-Induced Barrier Disruption. Nutrients.

[176] Aguilar E.C., Leonel A.J., Teixeira L.G., Silva A.R., Silva J.F., Pelaez J.M.N., Capettini L.S.A., Lemos V.S., Santos R.A.S., Alvarez-Leite J.I. (2014). Butyrate Impairs Atherogenesis by Reducing Plaque Inflammation and Vulnerability and Decreasing NFκB Activation. Nutr. Metab. Cardiovasc. Dis..

[177] Hoffelner D.K., Hendrikx T. (2025). Emerging Therapy Targets to Modulate Microbiome-Mediated Effects Evident in Cardiovascular Disease. Front. Cardiovasc. Med..

[178] Mueller N.T., Zhang M., Juraschek S.P., Miller E.R., Appel L.J. (2020). Effects of High-Fiber Diets Enriched with Carbohydrate, Protein, or Unsaturated Fat on Circulating Short Chain Fatty Acids: Results from the OmniHeart Randomized Trial. Am. J. Clin. Nutr..

[179] Chakaroun R.M., Olsson L.M., Bäckhed F. (2023). The Potential of Tailoring the Gut Microbiome to Prevent and Treat Cardiometabolic Disease. Nat. Rev. Cardiol..

[180] Schiattarella G.G., Sannino A., Toscano E., Giugliano G., Gargiulo G., Franzone A., Trimarco B., Esposito G., Perrino C. (2017). Gut Microbe-Generated Metabolite Trimethylamine-N-Oxide as Cardiovascular Risk Biomarker: A Systematic Review and Dose-Response Meta-Analysis. Eur. Heart J..

[181] Gregory J.C., Buffa J.A., Org E., Wang Z., Levison B.S., Zhu W., Wagner M.A., Bennett B.J., Li L., DiDonato J.A. (2015). Transmission of Atherosclerosis Susceptibility with Gut Microbial Transplantation. J. Biol. Chem..

[182] Koeth R.A., Wang Z., Levison B.S., Buffa J.A., Org E., Sheehy B.T., Britt E.B., Fu X., Wu Y., Li L. (2013). Intestinal Microbiota Metabolism of L-Carnitine, a Nutrient in Red Meat, Promotes Atherosclerosis. Nat. Med..

[183] Farhangi M.A., Vajdi M. (2020). Novel Findings of the Association between Gut Microbiota–Derived Metabolite Trimethylamine *N-* Oxide and Inflammation: Results from a Systematic Review and Dose-Response Meta-Analysis. Crit. Rev. Food Sci. Nutr..

[184] Wang Z., Roberts A.B., Buffa J.A., Levison B.S., Zhu W., Org E., Gu X., Huang Y., Zamanian-Daryoush M., Culley M.K. (2015). Non-Lethal Inhibition of Gut Microbial Trimethylamine Production for the Treatment of Atherosclerosis. Cell.

[185] Roberts A.B., Gu X., Buffa J.A., Hurd A.G., Wang Z., Zhu W., Gupta N., Skye S.M., Cody D.B., Levison B.S. (2018). Development of a Gut Microbe–Targeted Nonlethal Therapeutic to Inhibit Thrombosis Potential. Nat. Med..

[186] Von Eckardstein A., Binder C.J. (2022). Prevention and Treatment of Atherosclerosis: Improving State-of-the-Art Management and Search for Novel Targets. Handbook of Experimental Pharmacology.

[187] Charach G., Argov O., Geiger K., Charach L., Rogowski O., Grosskopf I. (2018). Diminished Bile Acids Excretion Is a Risk Factor for Coronary Artery Disease: 20-Year Follow up and Long-Term Outcome. Ther. Adv. Gastroenterol..

[188] Tveter K.M., Mezhibovsky E., Wu Y., Roopchand D.E. (2023). Bile Acid Metabolism and Signaling: Emerging Pharmacological Targets of Dietary Polyphenols. Pharmacol. Ther..

[189] Hukkanen J., Küblbeck J., Hakkola J., Rysä J. (2025). Nuclear Receptors CAR and PXR as Cardiometabolic Regulators. Pharmacol. Res..

[190] Kriaa A., Bourgin M., Potiron A., Mkaouar H., Jablaoui A., Gérard P., Maguin E., Rhimi M. (2019). Microbial Impact on Cholesterol and Bile Acid Metabolism: Current Status and Future Prospects. J. Lipid Res..

[191] Juste C., Gérard P. (2021). Cholesterol-to-Coprostanol Conversion by the Gut Microbiota: What We Know, Suspect, and Ignore. Microorganisms.

[192] Kenny D.J., Plichta D.R., Shungin D., Koppel N., Hall A.B., Fu B., Vasan R.S., Shaw S.Y., Vlamakis H., Balskus E.P. (2020). Cholesterol Metabolism by Uncultured Human Gut Bacteria Influences Host Cholesterol Level. Cell Host Microbe.

[193] Yuan C., Yu B., Li L., Chen J., Qin W., Zhou Z., Su M., Wang D., Zhang Y., Wu Q. (2024). SUCNR 1 Promotes Atherosclerosis by Inducing Endoplasmic Reticulum Stress Mediated ER-Mito Crosstalk. Int. Immunopharmacol..

[194] Wei Y., Ma X., Zhao J., Wang X., Gao C. (2023). Succinate Metabolism and Its Regulation of Host-Microbe Interactions. Gut Microbes.

[195] Koh A., Mannerås-Holm L., Yunn N.-O., Nilsson P.M., Ryu S.H., Molinaro A., Perkins R., Smith J.G., Bäckhed F. (2020). Microbial Imidazole Propionate Affects Responses to Metformin through P38γ-Dependent Inhibitory AMPK Phosphorylation. Cell Metab..

[196] Seo S.-K., Kwon B. (2023). Immune Regulation through Tryptophan Metabolism. Exp. Mol. Med..

[197] Gupta S.K., Vyavahare S., Duchesne Blanes I.L., Berger F., Isales C., Fulzele S. (2023). Microbiota-Derived Tryptophan Metabolism: Impacts on Health, Aging, and Disease. Exp. Gerontol..

[198] Chajadine M., Laurans L., Radecke T., Mouttoulingam N., Al-Rifai R., Bacquer E., Delaroque C., Rytter H., Bredon M., Knosp C. (2024). Harnessing Intestinal Tryptophan Catabolism to Relieve Atherosclerosis in Mice. Nat. Commun..

[199] Xue H., Chen X., Yu C., Deng Y., Zhang Y., Chen S., Chen X., Chen K., Yang Y., Ling W. (2022). Gut Microbially Produced Indole-3-Propionic Acid Inhibits Atherosclerosis by Promoting Reverse Cholesterol Transport and Its Deficiency Is Causally Related to Atherosclerotic Cardiovascular Disease. Circ. Res..

[200] Jie Z., Xia H., Zhong S.-L., Feng Q., Li S., Liang S., Zhong H., Liu Z., Gao Y., Zhao H. (2017). The Gut Microbiome in Atherosclerotic Cardiovascular Disease. Nat. Commun..

[201] Koren O., Spor A., Felin J., Fåk F., Stombaugh J., Tremaroli V., Behre C.J., Knight R., Fagerberg B., Ley R.E. (2011). Human Oral, Gut, and Plaque Microbiota in Patients with Atherosclerosis. Proc. Natl. Acad. Sci. USA.

[202] Sayols-Baixeras S., Dekkers K.F., Baldanzi G., Jönsson D., Hammar U., Lin Y.-T., Ahmad S., Nguyen D., Varotsis G., Pita S. (2023). *Streptococcus* Species Abundance in the Gut Is Linked to Subclinical Coronary Atherosclerosis in 8973 Participants From the SCAPIS Cohort. Circulation.

[203] Li Y.-L., Chen B.-Y., Feng Z.-H., Zhou L.-J., Liu T., Lin W.-Z., Zhu H., Xu S., Bai X.-B., Meng X.-Q. (2025). Roles of Oral and Gut Microbiota in Acute Myocardial Infarction. J. Adv. Res..

[204] Ruan Q., Guan P., Qi W., Li J., Xi M., Xiao L., Zhong S., Ma D., Ni J. (2023). Porphyromonas Gingivalis Regulates Atherosclerosis through an Immune Pathway. Front. Immunol..

[205] Xie H., Qin Z., Ling Z., Ge X., Zhang H., Guo S., Liu L., Zheng K., Jiang H., Xu R. (2023). Oral Pathogen Aggravates Atherosclerosis by Inducing Smooth Muscle Cell Apoptosis and Repressing Macrophage Efferocytosis. Int. J. Oral Sci..

[206] Afzoon S., Amiri M.A., Mohebbi M., Hamedani S., Farshidfar N. (2023). A Systematic Review of the Impact of Porphyromonas Gingivalis on Foam Cell Formation: Implications for the Role of Periodontitis in Atherosclerosis. BMC Oral Health.

[207] Zhang T., Kurita-Ochiai T., Hashizume T., Du Y., Oguchi S., Yamamoto M. (2010). *Aggregatibacter Actinomycetemcomitans* Accelerates Atherosclerosis with an Increase in Atherogenic Factors in Spontaneously Hyperlipidemic Mice. FEMS Immunol. Med. Microbiol..

[208] Ghosh T.S., Valdes A.M. (2023). Evidence for Clinical Interventions Targeting the Gut Microbiome in Cardiometabolic Disease. BMJ.

[209] Yuan L., Li Y., Chen M., Xue L., Wang J., Ding Y., Gu Q., Zhang J., Zhao H., Xie X. (2024). Therapeutic Applications of Gut Microbes in Cardiometabolic Diseases: Current State and Perspectives. Appl. Microbiol. Biotechnol..

[210] O’Keefe S.J. (2019). The Association between Dietary Fibre Deficiency and High-Income Lifestyle-Associated Diseases: Burkitt’s Hypothesis Revisited. Lancet Gastroenterol. Hepatol..

[211] Burkitt D.P. (1969). Related Disease—Related Cause?. Lancet.

[212] Burkitt D. (1970). Relationship as a Clue to Causation. Lancet.

[213] Jing L., Zhang H., Xiang Q., Shen L., Guo X., Zhai C., Hu H. (2022). Targeting Trimethylamine N-Oxide: A New Therapeutic Strategy for Alleviating Atherosclerosis. Front. Cardiovasc. Med..

[214] Reynolds A.N., Akerman A.P., Mann J. (2020). Dietary Fibre and Whole Grains in Diabetes Management: Systematic Review and Meta-Analyses. PLoS Med..

[215] Barber T.M., Kabisch S., Pfeiffer A.F.H., Weickert M.O. (2020). The Health Benefits of Dietary Fibre. Nutrients.

[216] Xiao J.-H., Wang Y., Zhang X.-M., Wang W.-X., Zhang Q., Tang Y.-P., Yue S.-J. (2024). Intestinal Permeability in Human Cardiovascular Diseases: A Systematic Review and Meta-Analysis. Front. Nutr..

[217] Campbell C.D., Gleeson M., Sulaiman I. (2023). The Role of the Respiratory Microbiome in Asthma. Front. Allergy.

[218] Valverde-Molina J., García-Marcos L. (2023). Microbiome and Asthma: Microbial Dysbiosis and the Origins, Phenotypes, Persistence, and Severity of Asthma. Nutrients.

[219] Roduit C., Frei R., Ferstl R., Loeliger S., Westermann P., Rhyner C., Schiavi E., Barcik W., Rodriguez-Perez N., Wawrzyniak M. (2019). High Levels of Butyrate and Propionate in Early Life Are Associated with Protection against Atopy. Allergy.

[220] Facchin S., Bertin L., Bonazzi E., Lorenzon G., De Barba C., Barberio B., Zingone F., Maniero D., Scarpa M., Ruffolo C. (2024). Short-Chain Fatty Acids and Human Health: From Metabolic Pathways to Current Therapeutic Implications. Life.

[221] Violi F., Cammisotto V., Bartimoccia S., Pignatelli P., Carnevale R., Nocella C. (2023). Gut-Derived Low-Grade Endotoxaemia, Atherothrombosis and Cardiovascular Disease. Nat. Rev. Cardiol..

[222] Bagheri B., Khatibiyan Feyzabadi Z., Nouri A., Azadfallah A., Mahdizade Ari M., Hemmati M., Darban M., Alavi Toosi P., Banihashemian S.Z. (2024). Atherosclerosis and Toll-Like Receptor4 (TLR4), Lectin-Like Oxidized Low-Density Lipoprotein-1 (LOX-1), and Proprotein Convertase Subtilisin/Kexin Type9 (PCSK9). Mediat. Inflamm..

[223] Li X., Wang Y., Xu J., Luo K., Dong T. (2024). Association between Trimethylamine N-Oxide and Prognosis of Patients with Myocardial Infarction: A Meta-Analysis. Front. Cardiovasc. Med..

[224] Losol P., Wolska M., Wypych T.P., Yao L., O’Mahony L., Sokolowska M. (2024). A Cross Talk between Microbial Metabolites and Host Immunity: Its Relevance for Allergic Diseases. Clin. Transl. Allergy.

[225] Zhang H., Dhalla N.S. (2024). The Role of Pro-Inflammatory Cytokines in the Pathogenesis of Cardiovascular Disease. Int. J. Mol. Sci..

[226] Wolf D., Ley K. (2019). Immunity and Inflammation in Atherosclerosis. Circ. Res..

[227] Kral M., Van Der Vorst E.P.C., Surnov A., Weber C., Döring Y. (2023). ILC2-Mediated Immune Crosstalk in Chronic (Vascular) Inflammation. Front. Immunol..

[228] Kim H.R., Ingram J.L., Que L.G. (2023). Effects of Oxidative Stress on Airway Epithelium Permeability in Asthma and Potential Implications for Patients with Comorbid Obesity. J. Asthma Allergy.

[229] Van Beveren G.J., Said H., Van Houten M.A., Bogaert D. (2023). The Respiratory Microbiome in Childhood Asthma. J. Allergy Clin. Immunol..

[230] Razeghian-Jahromi I., Elyaspour Z., Zibaeenezhad M.J., Hassanipour S. (2022). Prevalence of Microorganisms in Atherosclerotic Plaques of Coronary Arteries: A Systematic Review and Meta-Analysis. Evid.-Based Complement. Altern. Med..

[231] El-saadawi A.A., Hafez M.R., Ibrahim R.S., Eid H.A., Sakr L.K. (2024). Study of Atherosclerosis in Bronchial Asthma Patients. J. Recent Adv. Med..

[232] Menegati L.M., De Oliveira E.E., Oliveira B.D.C., Macedo G.C., De Castro E Silva F.M. (2023). Asthma, Obesity, and Microbiota: A Complex Immunological Interaction. Immunol. Lett..

[233] Zhang X., Li J., Luo S., Wang M., Huang Q., Deng Z., De Febbo C., Daoui A., Liew P.X., Sukhova G.K. (2020). IgE Contributes to Atherosclerosis and Obesity by Affecting Macrophage Polarization, Macrophage Protein Network, and Foam Cell Formation. Arterioscler. Thromb. Vasc. Biol..

[234] Zimbru E.-L., Zimbru R.-I., Ordodi V.-L., Bojin F.-M., Crîsnic D., Andor M., Mirica S.-N., Huțu I., Tănasie G., Haidar L. (2024). Rosuvastatin Attenuates Vascular Dysfunction Induced by High-Fructose Diets and Allergic Asthma in Rats. Nutrients.

[235] Wu J., He S., Song Z., Chen S., Lin X., Sun H., Zhou P., Peng Q., Du S., Zheng S. (2023). Macrophage Polarization States in Atherosclerosis. Front. Immunol..

[236] Haidar L., Bănărescu C.F., Uța C., Zimbru E.-L., Zimbru R.-I., Tîrziu A., Pătrașcu R., Șerb A.-F., Georgescu M., Nistor D. (2025). Beyond the Skin: Exploring the Gut–Skin Axis in Chronic Spontaneous Urticaria and Other Inflammatory Skin Diseases. Biomedicines.

[237] Aldriwesh M.G., Al-Mutairi A.M., Alharbi A.S., Aljohani H.Y., Alzahrani N.A., Ajina R., Alanazi A.M. (2023). Paediatric Asthma and the Microbiome: A Systematic Review. Microorganisms.

[238] Wang H., He Y., Dang D., Zhao Y., Zhao J., Lu W. (2024). Gut Microbiota-Derived Tryptophan Metabolites Alleviate Allergic Asthma Inflammation in Ovalbumin-Induced Mice. Foods.

[239] Kazemian N., Mahmoudi M., Halperin F., Wu J.C., Pakpour S. (2020). Gut Microbiota and Cardiovascular Disease: Opportunities and Challenges. Microbiome.

[240] Tang W.H.W., Wang Z., Levison B.S., Koeth R.A., Britt E.B., Fu X., Wu Y., Hazen S.L. (2013). Intestinal Microbial Metabolism of Phosphatidylcholine and Cardiovascular Risk. N. Engl. J. Med..

[241] Nemet I., Saha P.P., Gupta N., Zhu W., Romano K.A., Skye S.M., Cajka T., Mohan M.L., Li L., Wu Y. (2020). A Cardiovascular Disease-Linked Gut Microbial Metabolite Acts via Adrenergic Receptors. Cell.

[242] Fu Y., Yang Y., Fang C., Liu X., Dong Y., Xu L., Chen M., Zuo K., Wang L. (2022). Prognostic Value of Plasma Phenylalanine and Gut Microbiota-Derived Metabolite Phenylacetylglutamine in Coronary in-Stent Restenosis. Front. Cardiovasc. Med..

[243] Zheng X., Chen M., Zhuang Y., Zhao L., Qian Y., Shi C. (2024). Unveiling Genetic Links between Gut Microbiota and Asthma: A Mendelian Randomization. Front. Microbiol..

[244] Ramar M., Wiscovitch-Russo R., Yano N., Singh H., Lamere E., Short M., Gonzalez-Juarbe N., Fedulov A.V. (2025). Live Bacteria in Gut Microbiome Dictate Asthma Onset Triggered by Environmental Particles via Modulation of DNA Methylation in Dendritic Cells. Cell Rep..

[245] Zhu Y., Li Q., Jiang H. (2020). Gut Microbiota in Atherosclerosis: Focus on Trimethylamine N-oxide. APMIS.

[246] Li X., Su C., Jiang Z., Yang Y., Zhang Y., Yang M., Zhang X., Du Y., Zhang J., Wang L. (2021). Berberine Attenuates Choline-Induced Atherosclerosis by Inhibiting Trimethylamine and Trimethylamine-N-Oxide Production via Manipulating the Gut Microbiome. Npj Biofilms Microbiomes.

[247] Pala B., Tocci G., Nardoianni G., Barbato E., Amedei A. (2024). Gut Microbiome and Carotid Artery Intima-Media Thickness: A Narrative Review of the Current Scenario. Diagnostics.

[248] Jarmukhanov Z., Mukhanbetzhanov N., Kozhakhmetov S., Nurgaziyev M., Sailybayeva A., Bekbossynova M., Kushugulova A. (2024). The Association between the Gut Microbiota Metabolite Trimethylamine N-Oxide and Heart Failure. Front. Microbiol..

[249] Zhou Y., Zhang Y., Jin S., Lv J., Li M., Feng N. (2024). The Gut Microbiota Derived Metabolite Trimethylamine N-Oxide: Its Important Role in Cancer and Other Diseases. Biomed. Pharmacother..

[250] Ye F., Li L., Wang J., Yang H. (2025). Advances in Gut-Lung Axis Research: Clinical Perspectives on Pneumonia Prevention and Treatment. Front. Immunol..

[251] Abdulrahim A.O., Doddapaneni N.S.P., Salman N., Giridharan A., Thomas J., Sharma K., Abboud E., Rochill K., Shreelakshmi B., Gupta V. (2025). The Gut–Heart Axis: A Review of Gut Microbiota, Dysbiosis, and Cardiovascular Disease Development. Ann. Med. Surg..

